# EvoSNR-Prom: Predicting promoters at single-nucleotide resolution with label-aware transfer learning of the pretrained EVO model

**DOI:** 10.1371/journal.pcbi.1014626

**Published:** 2026-08-10

**Authors:** Pi-Jing Wei, Wenkang Zheng, Yijun Gu, Chun-Hou Zheng

**Affiliations:** 1 Institutes of Physical Science and Information Technology, Anhui University, Hefei, Anhui, China; 2 Information Materials and Intelligent Sensing Laboratory of Anhui Province, Anhui University, Hefei, Anhui, China; 3 School of Artificial Intelligence, Anhui University, Hefei, Anhui, China; The Pennsylvania State University, UNITED STATES OF AMERICA

## Abstract

The precise identification of promoters is crucial for understanding gene regulation. Deep learning methods have achieved considerable success in promoter prediction, yet most operate at the sequence level with coarse-grained labels. This means they label an entire DNA segment as either a “promoter” or “non-promoter,” which results in a lack of the nucleotide-level resolution in prediction. In this study, we propose EvoSNR-Prom, a model designed for promoter prediction at single-nucleotide resolution. EvoSNR-Prom is built on the Evo foundation model and formulates promoter identification as a token-level sequence labeling problem, analogous to named entity recognition in natural language processing. To address the limited contextual information available in single-nucleotide tokenization, we introduce a lexicon-enhanced embedding strategy that incorporates biologically meaningful DNA lexicons, enriching contextual representations and improving the model’s ability to capture complex sequence motifs. Furthermore, to enhance predictive performance on small size datasets, we integrate a label-aware transfer learning framework to leverage knowledge from well-annotated source species to a target organism. The results across various prokaryotic datasets show that EvoSNR-Prom achieves excellent performance. This work provides a valuable computational framework for the high-precision analysis of gene regulatory elements, contributing to the advancement of promoter prediction at single-nucleotide resolution.

## Author summary

Understanding how genes are turned on and off is a central question in biology. Promoters, which are short regions of DNA that control gene activity, play central role in this process. Accurate identification of promoters is essential for understanding gene regulatory mechanisms. While deep learning methods have driven significant progress in promoter prediction, the majority of existing models operate at a coarse-grained sequence level. Most of them work at a coarse level: they treat long stretches of DNA as either “promoter” or “non-promoter.” This window-based view misses finer-resolution information within promoter regions. In this study, we introduce EvoSNR-Prom, a new approach that predicts promoters at single-nucleotide resolution. Instead of labeling entire DNA segments, our method examines each nucleotide one by one, enabling finer-resolution prediction. We treat DNA sequences as a language, drawing inspiration from tasks like named entity recognition (NER). This perspective allows us to combine a powerful DNA foundation model with biologically meaningful lexicons, enabling more effective capture of complex sequence patterns. To further enhance performance, our framework also transfers knowledge between species, enabling accurate promoter prediction even in less well-studied organisms. EvoSNR-Prom represents a promising step toward high-resolution analysis of gene regulation. This view may help researchers uncover subtle regulatory signals that were previously overlooked, offering new opportunities for biological discovery and applications in biotechnology.

## Introduction

Gene expression is a central process in all life, and its precise regulation is fundamental to a cell’s ability to respond to internal and external environmental changes and maintain homeostasis. Within this complex regulatory mechanism, promoters which are located upstream of the Transcription Start Site (TSS) play a central role in regulation of gene expression. Promoters function by interacting with RNA polymerase and its accessory factors (such as sigma factors) through specific DNA sequence motifs, which dictates the timing, strength, and specificity of transcription [1–5]. The accurate prediction of promoters plays a pivotal role in understanding the mechanisms of gene regulation.

Over the past several decades, promoter prediction has evolved from rule-based statistical approaches to classical machine learning methods and, more recently, to advanced deep learning architectures. Conceptually, the Transcription Start Site (TSS) serves as a key reference point for defining the promoter region. Consequently, early approaches usually focused on identifying conserved sequence motifs associated with the TSS. For example, TSSW and PromH [6,7] employed Linear Discriminant Analysis (LDA) to integrate predefined motif-based rules with local sequence statistics for TSS localization [8]. In these frameworks, the predicted TSS often serves as a reference point for subsequent characterization of promoter-associated regions. In parallel, Hidden Markov Model (HMM)-based approaches provided a complementary probabilistic framework capable of capturing the transitional biological properties inherent to promoter regions [9,10]. While these statistical and probabilistic frameworks established an important foundation for nucleotide-resolution promoter analysis, their performance was often constrained by a heavy reliance on highly conserved sequence signals.

To overcome the limitations of relying solely on motifs, researchers adopted classical machine learning, which emphasized feature engineering-manually extracting informative biological features from DNA sequences. By training classifiers on these curated features, models could recognize more complex promoter patterns, substantially improving predictive performance over earlier methods. Representative examples include iPro54-PseKNC [11] and 70ProPred [12], which leverage support vector machines (SVM) combined with sequence-derived features like pseudo *k*-tuple nucleotide composition (PseKNC) to classify promoter regions. Nevertheless, the performance of these methods remains dependent on the quality and completeness of manually engineered features. The emergence of deep learning has alleviated this limitation by its exceptional capability in automatic feature representation, leading to superior performance in promoter prediction. Early advances were driven mainly by Convolutional Neural Networks (CNNs) and Recurrent Neural Networks (RNNs)—for example, DeePromoter [13], CNNProm [14], DeeReCT-PromID [15], DeeProPre [16]. By learning hierarchical, task-specific representations directly from raw nucleotide sequences, these models markedly outperformed rule-based or shallow statistical algorithms and removed the need for manual feature engineering. Nevertheless, CNN- and RNN-based architectures are constrained in modeling long-range nucleotide dependencies due to their limited receptive fields and sequential processing nature, respectively. To address this constraint, Transformer-based models, built upon the attention mechanism, have been employed. They can dynamically weigh the importance of relationships between any two nucleotides in a sequence, regardless of their distance. This allows it to capture complex, long-range regulatory grammar more flexibly and effectively. A widely adopted strategy is to fine-tune pre-trained DNA foundation models, such as DNABERT [17] or the Nucleotide Transformer [18], by adding task-specific classification heads. Despite their success, current deep learning models are still primarily designed for coarse-grained, sequence-level classification.

High-resolution sequence annotation paradigm has been successfully applied to the fine-mapping of transcription factor binding sites (TFBS). State-of-the-art models such as DeepSNR [19], D-AEDNet [20], and BertSNR [21] have demonstrated the ability to predict transcription factor binding intensity at single-nucleotide resolution by leveraging high-resolution experimental data. Their output is not a merely a coarse sequence-level TFBS/non-TFBS label but rather a profile of single-nucleotide predictions along the DNA sequence. This methodological success provides compelling evidence that deep learning models are capable of learning and decoding fine-grained, single-nucleotide regulatory signals directly from sequence data. In contrast, models specifically designed for single-nucleotide-level promoter prediction remain relatively limited. Recent general-purpose genome annotation models provide a potential alternative. For example, SegmentNT [22], frames genome annotation as a nucleotide-level multi-class segmentation problem and predicts 14 types of genomic elements, including promoters. Nucleotide Transformer v3 (NTv3) [23] is the latest iteration of the Nucleotide Transformer [18] series. It extends its training to a broader range of species, yielding further improvements in genomic prediction. Nevertheless, these models are primarily optimized for general genome annotation and functional prediction rather than promoter-specific, nucleotide-resolution modeling.

In this study, we propose EvoSNR-Prom, a single-nucleotide-resolution model with lexicon enhancement and transfer learning strategy for prokaryotic promoter prediction. It builds on Evo [24] pre-trained foundation model,whose StripedHyena architecture [25,26] facilitates efficient processing of contexts up to 131 kb at byte-level nucleotide resolution, thereby preserving the fine-grained detail indispensable for promoter prediction at single-nucleotide resolution. Furthermore, its expansive pretraining dataset—encompassing 2.7 million prokaryotic and phage genomes—and inherent multimodality across DNA, RNA, and protein sequences render Evo an ideal foundation model for this task. However, while single-nucleotide tokenization is crucial for fine-grained resolution, it can lead to the loss of semantic information embedded in larger motifs. To mitigate this, we introduce a lexicon enhancement strategy. By incorporating externally curated DNA lexicons as auxiliary knowledge, EvoSNR-Prom gains explicit lexical cues, enabling more precise delineation of entity spans and more accurate semantic type assignments. This not only enhances sequence representations but also mitigates data sparsity. Furthermore, to address the scarcity of annotated data, label-aware transfer learning is adopted to enhance model performance. Evaluated across a diverse set of prokaryotic species, EvoSNR-Prom achieves high-precision promoter prediction at single-nucleotide resolution. This study also paves the way for the prediction and design of DNA regulatory elements at single-nucleotide resolution in the future research.

## Materials and methods

### Dataset

The promoter dataset for training and testing was compiled from the Prokaryotic Promoter Database (PPD) [27], a public repository of experimentally validated promoters. The primary criterion for inclusion in PPD is that each promoter’s Transcription Start Site (TSS) must be supported by direct experimental evidence, which guarantees the biological authenticity and functional relevance of the sequences. This database is manually curated, with all entries extracted and annotated by domain experts from peer-reviewed literature, thus mitigating the systematic biases inherent in automated data-collection pipelines. However, despite the quality of comprehensive databases like PPD, the availability of experimentally validated promoter data is highly uneven across the vast spectrum of prokaryotic species. Many organisms, particularly non-model species, lack sufficient labeled examples to train robust predictive models from scratch. To address this challenge of data scarcity, we adopted a transfer learning approach. It is crucial for transfer learning to maintain relatedness between the source and target domains. To meet this prerequisite, in our model, the TimeTree5 [28] tool was employed to estimate evolutionary divergence times among candidate prokaryotic species in the PPD (S1 Appendix), which provided the basis for constructing source–target domain pairs. Based on these estimates, two source–target domain pairs were constructed: (i) *Sinorhizobium meliloti* 1021 (source domain) and *Agrobacterium tumefaciens* strain C58 (target domain), and (ii) *Escherichia coli* str. K-12 substr. MG1655 (source domain) and *Klebsiella aerogenes* KCTC 2190 (target domain). The selection of these species pairs was determined by their relatively recent divergence from a common ancestor, thereby ensuring sufficient phylogenetic relatedness for effective transfer learning while preserving inter-species variability for rigorous evaluation.

Dataset preparation involved the systematic extraction of 500 bp DNA fragments. For each positive sample, a 500 bp genomic fragment was extracted around the 81 bp core promoter region annotated in the Prokaryotic Promoter Database (PPD) [27]. The promoter was assigned a random offset within the fragment, ensuring its complete inclusion while introducing variability in the upstream and downstream flanking sequences. The input length was set to 500 bp because shorter sequences may provide limited positional variation and insufficient contextual information, whereas longer sequences may dilute the promoter signal with excessive non-promoter background. An equal number of synthetic negative samples were generated using ushuffle [29], which shuffles positive sequences while preserving dinucleotide frequencies. Following sequence extraction, each nucleotide was assigned a binary label: ‘1’ for positions within the promoter region and ‘0’ for all others. Finally, for each species, the dataset was partitioned into training (80%), validation (10%), and testing (10%) subsets. The validation set was used for hyperparameter tuning and model selection, while the test set was reserved for final performance evaluation.

### EvoSNR-Prom framework

**Fig 1:**
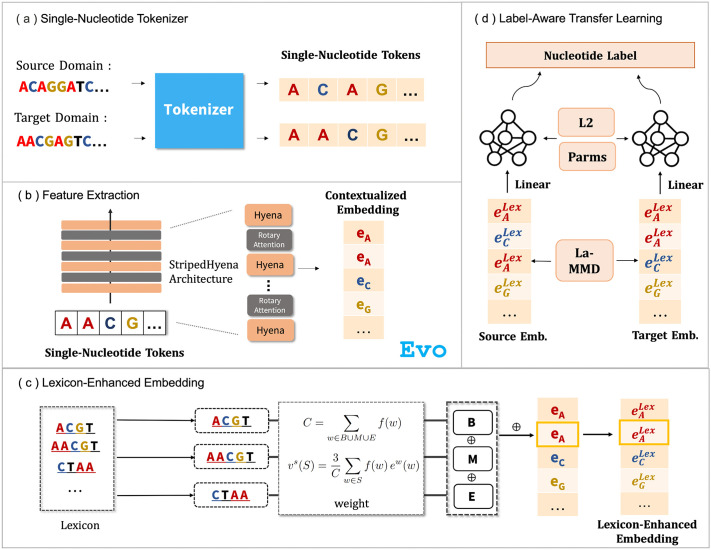
EvoSNR-Prom Model overview. **(a)** The input source and target domain DNA sequences are processed by single-nucleotide tokenizer, treating each nucleotide as a token. **(b)** The tokenized DNA sequences are fed into the Evo pre-trained model. **(c)** We apply a lexical enhancement to each contextualized embedding by integrating weighted motif embeddings derived from a motif lexicon, where the weights encode motif frequency and positional relevance. **(d)** The embeddings from both domains are adapted using label-aware transfer learning.

Our proposed deep learning model, EvoSNR-Prom, is built upon Evo [24], a powerful genomics foundation model pre-trained on a vast dataset of prokaryotic whole genomes encompassing over 300 billion tokens. The pre-training was performed at single-nucleotide resolution, which endows Evo with the ability to generate sequence contextualized embedding that are exquisitely sensitive to local context. Consequently, these embeddings capture not only long-range dependencies but also the fine-grained local patterns crucial for defining functional genetic elements. In this study, EvoSNR-Prom leverages this capability and is specifically designed to identify promoters with single-nucleotide precision. The architecture, illustrated in Fig 1, comprises four key modules: (a) Single-Nucleotide Tokenization, (b) Feature Extraction, (c) Lexicon-Enhanced Embedding, and (d) Label-Aware Transfer Learning.

### Single-nucleotide tokenizer

The primary objective of this study is the precise identification of prokaryotic promoter at single-nucleotide resolution. In the broader field of fine-grained genomic prediction, a prevalent strategy has been to leverage DNA language models that employ *k*-mer based tokenization. For instance, models like SegmentNT [22] and BertSNR [21] utilize *k*-mer tokenizers for genomic element segmentation and TFBS identification, respectively. Although effective for broader contexts, *k*-mer tokenization requires complex bidirectional mapping to resolve tokens back into single-nucleotide predictions [21]. This mapping process is prone to error propagation and collapses fine-grained positional signals [30,31]. Consequently, it creates an information bottleneck that limits the models’ capacity for single-base resolution prediction.

In contrast to prevalent approaches that segment DNA sequences into *k*-mers, our model leverages the native tokenizer of the Evo foundation model. Specifically, this is a ByteTokenizer, which processes the sequence at the individual character (byte) level. For DNA data, this ensures that every single nucleotide—’A,’ ‘C,’ ‘G,’ ‘T’—is mapped to a distinct token, thus preserving the highest possible resolution. The tokenizer employs a vocabulary of 512 tokens, which encompasses representations for all possible byte values as well as special-purpose tokens required by the underlying StripedHyena architecture. The output of this stage is a sequence of discrete tokens, providing an uncompressed, high-fidelity input for the subsequent layers of the model.

### Feature extraction

For feature extraction, we employ the pre-trained Evo model, a 7-billion-parameter autoregressive biological foundation model renowned for its ability to model long-range contextual dependencies in genomic data. Evo’s power is rooted in its StripedHyena [24,25,32]architecture, a hybrid deep signal processing framework that offers superior scalability over conventional decoder-only Transformers. Instead of standard self-attention, StripedHyena utilizes “Hyena blocks” that combine gated convolutions with a rotary attention mechanism. The fundamental component of this architecture is the Hyena operator, which functions as a recursive process driven by two core operations: an implicit long convolution and a data-controlled gating mechanism. The mathematical formulation of the Order–N Hyena Operator is given by:


$$\begin{array}{c} \begin{array}{c} z_{t}^{\text{1}}=v_{t} \end{array} \end{array}$$ (1)

$$\begin{array}{c} \begin{array}{c} z_{t}^{\left(n+\text{1}\right)}=x_{t}^{n}{\left(h^{n}*z^{n}\right)}_{t},n=\text{1},\ldots ,N \end{array} \end{array}$$ (2)

$$\begin{array}{c} \begin{array}{c} y_{t}=z_{t}^{\left(N+\text{1}\right)} \end{array} \end{array}$$ (3)

where $z_{t}^{n}$ represents the intermediate state of the sequence at time step t for the n-th step of the recurrence. The term $x_{t}^{n}$ denotes one of the N data-controlled gating projections derived from the input sequence. The symbol $h^{n}$ represents the learnable long convolutional filter specific to the n-th layer of the recurrence. The operator (*) signifies a discrete convolution, which is efficiently computed in the Fourier domain. $y_{t}$ represents the final output of the operator at a specific time step t in the sequence.

The Hyena operator’s computation is defined by a recursive process. The process commences by generating a set of N+1 linear projections from the input sequence. These projections are denoted as $\left(v,x^{\text{1}},\ldots ,x^{N}\right)$, where each element is a sequence of length L. Within this framework, the projection v serves as the initial state for the recurrence, while the projections $\left(x^{\text{1}},\ldots ,x^{N}\right)$ act as data-controlled multiplicative gates at each subsequent step. Equation 1 represents the initialization stage of the recurrent computational process defined within the Hyena operator. Following Equation (2), each step of the recursion involves two key operations: (1) Long Convolution: At each step n of the recurrence, an implicit long convolution is first performed between the state from the preceding step, $z^{n}$, and a learnable filter, $h^{n}$. This operation is designed to capture global contextual information and long-range dependencies across the input sequence. (2) Data-Controlled Gating: Subsequently, the output of the convolution is subjected to a data-controlled gating mechanism. This is executed as an element-wise multiplication of the convolved sequence with a corresponding gating sequence, $x^{n}$. This gating sequence is dynamically generated from a projection of the original input, allowing the model to selectively filter the convolved output and thereby modulate the information flow based on the input’s specific content. The result of this operation yields the updated state $z^{n+\text{1}}$ for the next iteration. The output $z_{t}^{\left(N+\text{1}\right)}$of this step then serves as the input for the subsequent iteration. By elegantly alternating between these operations which can be efficiently implemented in the frequency domain- the Hyena operator thus achieves performance comparable to or better than the attention mechanism. It effectively captures long-range dependencies while maintaining a sub-quadratic computational complexity.

By processing these sequences through Evo’s Hyena-based layers, we obtain contextualized embeddings at single-nucleotide resolution. This methodology is particularly powerful for genomics, as the sub-quadratic scaling permits the analysis of extensive genomic contexts, enabling the model to learn regulatory interactions that span tens of kilobases. The resulting vector representations are therefore deeply contextualized and informed by long-range dependencies, providing a robust foundation for accurately predicting complex biological phenomena directly from the primary DNA sequence.

### Lexicon-enhanced embedding

Single-nucleotide tokenization preserves maximal biological resolution but introduces a inherent representational challenge: individual nucleotides possess weak semantic value at the single-token level, and their functional significance is highly context-dependent rather than encapsulated within any isolated token [30]. Traditional *k*-mer tokenization can encode short local subsequences but blur exact nucleotide-level boundaries. To retain single-nucleotide resolution while explicitly supplying the missing local context, we propose a Lexicon Enhancement strategy. Inspired by lexicon-augmented character-level models in Chinese Named Entity Recognition (NER) [33–36], where individual characters are similarly underspecified and rely on word-level cues, we incorporate all relevant multi-nucleotide “DNA words” directly into the single-nucleotide representation layers. This design strengthens the representation of promoter-relevant local patterns and higher-order nucleotide dependencies without sacrificing single-nucleotide resolution or requiring substantial architectural changes.

Specifically, the process begins with the construction of a biologically meaningful DNA lexicon. To ensure functional relevance, we derived the lexicon from conserved motifs identified solely within the training set of promoter sequences. Here, the MEME Suite [37–39] was employed to perform *de novo* motif discovery across the dataset. Each high-confidence motif was treated as an individual lexicon term and encoded into a high-dimensional word vector representation. In this study, we adopted FastText [40] for motif encoding. Unlike standard lookup-based embeddings that assign one independent vector to each discrete motif token, FastText represents a motif as a composition of its character-level *n*-grams. This subword mechanism enables motifs with similar sequence composition to share representational components, thereby yielding a smoother embedding space that better reflects underlying motif relatedness [41]. In addition, subword-based parameter sharing can improve the representation of low-frequency motifs by leveraging recurrent local sequence patterns observed across the corpus. In this way, FastText naturally encodes the morphological proximity between functionally related motifs—a property that aligns well with the biological observation that motifs with high sequence similarity often exert comparable regulatory functions.

For single-nucleotide-resolution prediction, the model should be sensitive to each nucleotide’s relative position within a matched motif—whether it lies at the beginning, middle, or end. Naive fusion strategies such as averaging or max-pooling the embeddings of all lexicon-matched motifs at each nucleotide position would collapse this positional distinction into a uniform representation. To preserve this position-dependent information, we adopted the BMES (Begin–Middle–End–Single) tagging scheme from lexicon-augmented Chinese NER [35], which has been shown to significantly outperform position-agnostic fusion methods in sequence labeling tasks that require fine-grained positional discrimination. Specifically, we defined “BME” word sets to denote lexicon-matched motifs in which a given nucleotide occupies the beginning (B), middle (M), or end (E) position, respectively. By organizing matched motifs into these position-specific groups before fusion, the resulting representation encodes not only which motifs a nucleotide participates in, but also where within each motif the nucleotide is located. These structural cues directly enhance the model’s capacity to resolve functional elements at single-nucleotide resolution. To capture the distribution of local sequence patterns, we calculated the frequency of each term from our DNA lexicon across the training datasets. These frequencies subsequently guided a weighted fusion process, giving greater influence to more prevalent terms in the final lexicon-enhanced embedding [35]. Specifically, the weighted representation of the word set *S* is obtained as follows:


$$\begin{array}{c} v^{s}(S)=\frac{\text{3}}{c}\sum_{w\in S}f(w)e^{w}(w) \end{array}$$ (4)

$$\begin{array}{c} C=\sum_{w\in B\cup M\cup E}f(w) \end{array}$$ (5)

where S denotes a word set. For each word w∈S,its embedding $e^{w}$ is obtained by querying a pre-trained FastText model. f(w) denote the frequency that a lexicon word w occurs in the statistical data.

The last step involves integrating the embeddings from the three word sets into a single fixed-dimensional feature, which is then incorporated into each nucleotide character’s representation. We achieve this by concatenating the embeddings of the three word sets, resulting in the final representation for each nucleotide character $x^{c}$:


$$\begin{array}{c} \begin{array}{c} e^{s}(B,M,E)=\;\left[v^{s}(B);\;v^{s}(M);\;v^{s}(E)\right] \end{array} \end{array}$$ (6)

$$\begin{array}{c} \begin{array}{c} x^{c}\leftarrow \left[x^{c};\;e^{s}(B,M,E)\right] \end{array} \end{array}$$ (7)

We augment each nucleotide’s fundamental representation by fusing it with a rich, contextual vector derived from our DNA lexicon. This contextual vector is itself a composite, formed by concatenating the position-aware B, M, and E representations of matching lexicon motifs. This strategy effectively equips the model to simultaneously perceive both the fundamental ‘character’ (the nucleotide) and the contextual ‘words’ (the motifs), enabling a more nuanced and accurate interpretation of the DNA sequence.

### Label-aware transfer learning

The prediction of prokaryotic promoters is fundamentally challenged by a scarcity of annotated data, especially for less-studied organisms. To address this, we frame the problem as one of domain adaptation, enabling knowledge transfer from a well-annotated, label-rich source domain to a label-scarce target domain (a novel species). The underlying biological assumption is that while different prokaryotes may exhibit species-specific variations or “dialects” in their promoter sequences, they share a conserved structural “grammar.” Our model is architecturally designed to capture this relationship. In this study, we employ a shared feature extractor—composed of a finetuned Evo foundation model to learn these fundamental, domain-invariant representations. To translate these shared features into domain-specific predictions, the architecture then diverges into two domain-specific classifiers, this design allows the model to learn shared patterns while still providing the flexibility for each classifier to specialize in the unique characteristics of its respective domain. To bridge the domain gap, this is usually accomplished by minimizing the Maximum Mean Discrepancy (MMD) [42], which aligns the feature distributions of the two domains. MMD is a non-parametric metric that quantifies the distance between two probability distributions by comparing their mean embeddings in a high-dimensional Reproducing Kernel Hilbert Space (RKHS). A significant advantage of MMD is the utilization of the kernel trick, which enables the computation of distances in the RKHS without performing an explicit mapping of the samples. However, conventional MMD operates on a global level and risks negative transfer by mismatching features across different classes. To circumvent this, we utilize Label-aware MMD (La-MMD) [43], a variant that performs class-wise alignment.

La-MMD performs a class-wise feature distribution alignment by calculating the MMD for each label independently. For instance, it minimizes the distance between the distribution of ‘promoter’ samples from the source domain and that of the target domain, while also doing so for ‘non-promoter’ samples. This ensures a more precise alignment, preventing class confusion and enabling a more effective knowledge transfer. We define the La-MMD loss as:


$$\begin{array}{c} L_{La-MMD}=\sum_{c\in C}MMD^{\text{2}}\left(G\left(X_{s}^{\left(c\right)}\right),G\left(X_{t}^{\left(c\right)}\right)\right) \end{array}$$ (8)

where “C"  is the set of shared label classes between the source and target domains; $X_{s}^{\left(c\right)}$and $X_{t}^{\left(c\right)}$ are the subsets of samples in the source and target domains belonging to class c∈C, respectively; $G\left(X_{s}^{\left(c\right)}\right)$ and $G\left(X_{t}^{\left(c\right)}\right)$ represent the corresponding feature representations generated by the feature extractor G(·); and $MMD^{\text{2}}(\cdot ,\cdot )$ denotes the squared Maximum Mean Discrepancy, which measures the distance between the two distributions. By computing distributional distances separately within each label class and summing them, La-MMD ensures that only semantically equivalent representations are brought into correspondence, thereby preserving inter-class discriminability across domains.

In concert with feature-level adaptation, our framework introduces a second, complementary strategy at the classifier level. We hypothesize that the parameters of the well-trained source classifier contain valuable knowledge. Therefore, we introduce a Parameter Transfer Loss (denoted as $L_{param}$), which regularizes the distance between the weights of the target classifier and the source classifier. It effectively prevents the target classifier from overfitting on the limited target data by anchoring its solution to the robust knowledge base provided by the source. This entire learning process is encapsulated in our final objective function, which synergistically combines the supervised losses ($L_{s}\;,$${\;L}_{t}$), the La-MMD loss ($L_{La-MMD}\;$), the parameter loss ($L_{param}$), and a standard L2 regularization term ($L_{reg\;}$).


$$\begin{array}{c} {ℒ}_{total}=\;\left({ℒ}_{s}+\;{ℒ}_{t}\right)+\;\alpha \cdot {ℒ}_{La-MMD}+\;\beta \cdot {ℒ}_{param}+\;\gamma \;\cdot {ℒ}_{reg} \end{array}$$ (9)

where α,β,γ  are hyperparameters that balance the contribution of each term.

### Hyperparameters and implements

To train EvoSNR-Prom model, we employed a meticulously configured set of hyperparameters and a tailored training strategy to ensure effective knowledge transfer and strong generalization performance. We selected the AdamW optimizer and applied a differential learning rate scheme. Specifically, the pre-trained Evo feature extractor was fine-tuned with a conservative learning rate of 5e-5, whereas the newly initialized layers for the downstream task were trained with a higher learning rate of 2e-4 to facilitate faster convergence. The learning rate was managed by a linear scheduler with a warmup phase constituting the first 10% of total training steps, which stabilizes the initial training phase. To mitigate the challenge of data imbalance between the source and target domains, we implemented a custom dual-source balanced sampler. This sampler ensures that each batch contains an equal number of instances from the source and target domains. It is designed to iterate through the entire source domain dataset within each epoch while oversampling the smaller target domain dataset to match the required number of training steps. Furthermore, annotated boundaries in datasets do not necessarily correspond to precise strict biological boundaries. To accommodate this discrepancy, we optimized the model using a position-weighted loss function that assigns higher weights to core promoter regions and clear distant negatives, while significantly reducing the weight near annotated boundaries [44,45]. This strategy can reduce the impact of imprecise annotations and encourages the model to focus on biologically relevant promoter-associated features rather than strict boundary matching.

Given the substantial size of the Evo model, which contains 7 billion parameters, training on a single consumer-grade GPU is infeasible. To address this, we utilized Low-Rank Adaptation (LoRA) [46], a Parameter-Efficient Fine-Tuning (PEFT) [47] method. LoRA avoids updating the entire model by injecting trainable, low-rank matrices into the weight matrices of the pre-trained model. This approach dramatically reduces computational requirements while preserving the model’s original performance capabilities. In our implementation, LoRA was strategically applied to the stacked blocks of the Evo model. Specifically, LoRA configuration targeted key components for fine-tuning: the query-key-value (QKV) projection and output projection layers within the multi-head attention mechanism of specific blocks (8, 16, and 24), as well as the linear layers (l1 to l3) of the feed-forward networks (MLPs) across all 32 blocks. This targeted configuration, implemented using the PEFT library, ensures that only the low-rank adapters in these modules are updated. This LoRA setup reduced the number of trainable parameters from 7 billion to approximately 16 million—a reduction of ~99.77%—thereby enabling efficient training on a single NVIDIA RTX 4090 GPU with 24GB of VRAM. The application of LoRA significantly enhanced both training efficiency and overall model performance.

## Results

### Results and comparison

To the best of our knowledge, no prior work has specifically addressed single-nucleotide-resolution promoter prediction in prokaryotic genomes. To comprehensively evaluate the performance of EvoSNR-Prom, we benchmarked it against two task-specific architectures trained from scratch (DeepSNR [19] and D-AEDNet [20]) and two pre-trained genomic foundation models (BertSNR [21] and Nucleotide Transformer v3 [23]). All models were trained and evaluated on 500 bp input sequences.

To rigorously evaluate EvoSNR-Prom’s capabilities, we focused on its transfer learning performance across two target species chosen for their limited number of annotated promoters in the PPD [27] database: *Klebsiella aerogenes* KCTC 2190 (763 promoters) and *Agrobacterium tumefacien*s strain C58 (706 promoters). For each target, a phylogenetically close species was used as the source domain to enable transfer learning. Notably, while the competing models were trained exclusively on the target species’ promoters, EvoSNR-Prom leveraged combined source and target domain data.

**Fig 2:**
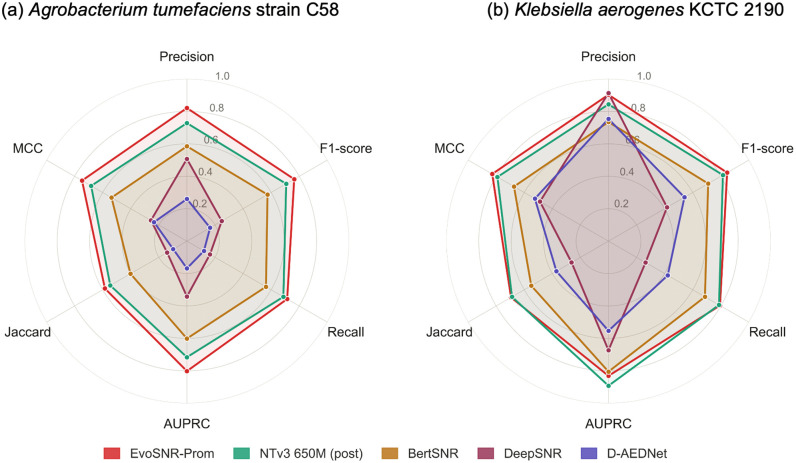
Performance comparison of EvoSNR-Prom and baseline models. Radar charts illustrate the evaluation results on **(a)** *Agrobacterium tumefaciens* strain C58 and **(b)** *Klebsiella aerogenes* KCTC 2190 datasets.

The results of comparing EvoSNR-Prom with the four competing models for single-nucleotide resolution promoter prediction are summarized in a radar chart (Fig 2), which provides a multi-dimensional visualization of performance across six key metrics: precision, recall, F1-score, AUPRC (Area Under the Precision–Recall Curve), Jaccard index, and MCC (Matthews Correlation Coefficient). The chart clearly illustrates that EvoSNR-Prom achieves the most balanced and competitive performance profile across both target species. Its performance polygon encloses the largest overall area, signifying consistent excellence across the majority of metrics rather than an isolated strength in specific ones. Among the baselines, NTv3 650M emerges as the strongest competitor, ranking second in most metrics and substantially outperforming the task-specific models, which suggests that large-scale pre-training may confer meaningful advantages for fine-grained genomic annotation. It is worth noting that NTv3 achieves a higher AUPRC (0.892) than EvoSNR-Prom (0.831) in *K. aerogenes* KCTC 2190, indicating a better overall precision–recall trade-off across different decision thresholds on this species. Detailed performance metrics for this comparison are available in S1 Table.

### Ablation study

To investigate the contribution of each component to the overall performance of EvoSNR-Prom, we conducted an ablation study on the *Agrobacterium tumefaciens* strain C58 dataset. This study was designed to quantify the impact of each key module by evaluating them against a Baseline model. This approach allows us to assess both the individual necessity of each component and their potential synergistic effects. Specifically, we first established a Baseline using the pre-trained Evo model as a feature extractor to generate context-aware representations from the input sequence. A Softmax classifier was then applied to these feature vectors to perform nucleotide-level functional classification. This model serves as a simple benchmark, as it lacks any architecture or optimization strategy tailored for the SNR task. Next, we independently augmented the Baseline with a Lexicon Enhancement Module (Baseline+Lexicon) and a Label-aware Transfer Learning Module (Baseline+Transfer Learning). Finally, we integrated both modules to construct the Full EvoSNR-Prom model.

The experimental results (Table 1) demonstrate that, compared with the baseline, the lexicon enhancement module increased the F1-score and MCC by over 2%. Similarly, independently integrating the transfer learning module increased the F1-score and MCC by over 3%. Ultimately, the complete EvoSNR-Prom model integrated both modules, leading to substantial improvements. Analyzing the ablation results from a domain perspective, the Baseline acts as a ‘target-only’ paradigm, whereas the Transfer Learning Module constitutes a ‘source+target’ approach. For a comprehensive comparison, we also evaluated a ‘source-only’ paradigm (S2 Table). The ‘source+target’ paradigm surpasses both counterparts across all metrics, validating the effectiveness of transfer learning.

**Table 1:** Ablation Study. Performance comparison of different variants of our model.

Method	Recall	F1-score	MCC	AUPRC
Baseline	70.27%	68.88%	66.09%	71.33%
Baseline + Lexicon	**73.95%**	71.16%	68.57%	70.73%
Baseline +Transfer learning	72.37%	72.69%	69.21%	72.03%
EvoSNR_Prom	71.40%	**76.38%**	**74.66%**	**79.21%**

### Transfer learning and domain adaptation analysis

To qualitatively assess how the label-aware transfer learning component influences the learned feature space, we visualized nucleotide-level embeddings using Uniform Manifold Approximation and Projection (UMAP) [48] on the *Agrobacterium tumefaciens* strain C58 dataset. The analysis was designed to examine two aspects: (1) the extent of distributional alignment between source- and target-domain representations, and (2) the class separability between promoter and non-promoter nucleotides. For this analysis, we extracted intermediate feature embeddings from the EvoEmb layer, the shared sequence-encoding module in our model that maps input nucleotide sequences into contextual feature representations. Comparing source and target embeddings at this layer is methodologically valid, as all embeddings are produced by the same representation function regardless of their domain origin.

**Fig 3:**
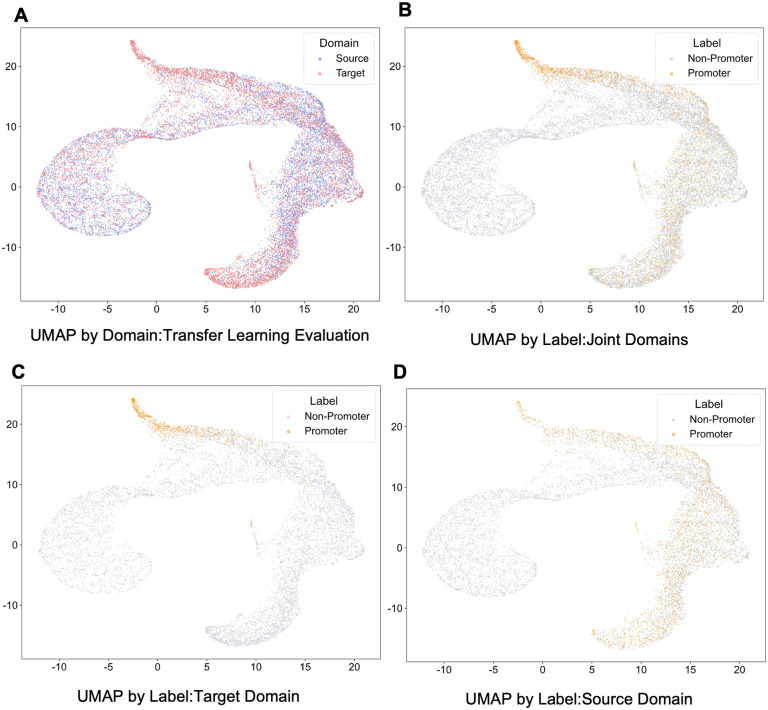
UMAP visualization of single-nucleotide embeddings. **(A)** UMAP projection of the transfer learning space colored by domain. **(B)** UMAP projection of the same space colored by true label. **(C)** Visualization of the target domain classification performance. **(D)** Visualization of the source domain classification performance.

The UMAP projections are presented in Fig 3. In Fig 3A, source and target embeddings are extensively intermingled rather than forming separate domain-specific clusters. This suggests that the label-aware transfer learning strategy may promote domain-invariant representations, which is beneficial for cross-species generalization. Fig 3B further shows that, within this mixed-domain manifold, promoter and non-promoter nucleotides form visually distinguishable groups, indicating that the shared embedding space retains class-discriminative structure when pooling source and target data. To assess class separation on each individual species, we visualize the embeddings for the target and source domains separately (Fig 3C-3D). The results demonstrate clear separation between promoter and non-promoter nucleotides within each respective embedding space.

### Attention and prediction visualization

**Fig 4:**
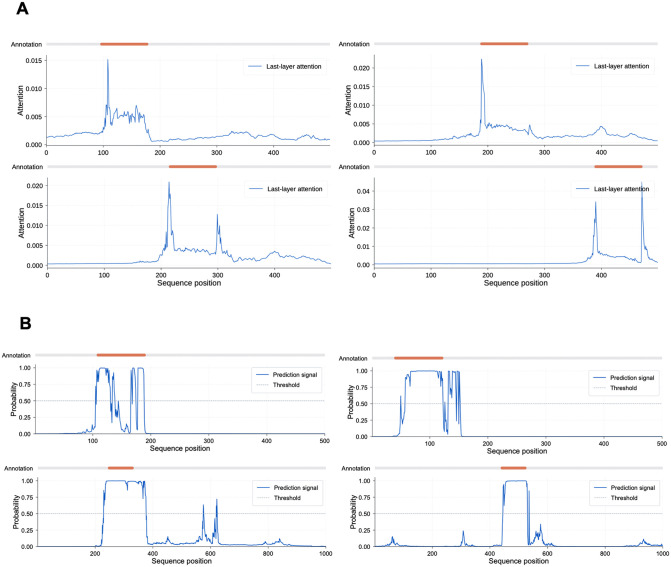
Attention and Prediction Visualization. In each panel, the orange bar at the top denotes the ground-truth annotated promoter region within the given sequence. **(A)** The last-layer attention weights across sequence positions for representative promoter sequences. **(B)** The prediction signals produced by the model. The 1000 bp prediction was implemented using a sliding-window scanning strategy.

To provide direct visual evidence of EvoSNR-Prom’s interpretability and predictive behavior, we conducted a two-part visualization analysis encompassing both the model’s internal attention patterns and its output prediction profiles (Fig 4). The attention analysis was used to assess which sequence positions are emphasized during representation learning, whereas the prediction-profile analysis was used to determine whether these internal patterns translate into spatially accurate promoter localization.

We first investigate the model’s internal attention behavior by extracting attention weight matrices from the Transformer layers of EvoSNR-Prom and summarizing them into position-wise attention profiles. Representative examples are shown in Fig 4A. Across four examples, in which the promoter region appears at different positions within the 500 bp input sequence, the attention profiles exhibit prominent peaks that spatially coincide with the annotated promoter regions. In contrast, flanking non-promoter positions receive comparatively lower attention. This spatial correspondence suggests that the model tends to allocate greater representational focus to regulatory regions rather than uniformly attending to the entire input sequence or relying primarily on flanking sequence context.

To determine whether these internal attention patterns are reflected in the model’s actual predictions, we further visualized the nucleotide-level output probabilities in Fig 4B. Specifically, each curve represents the per-position probability of belonging to the promoter class on representative test sequences. The upper row shows predictions on standard 500 bp input fragments. Across these examples, the predicted promoter probability rises sharply within the annotated promoter region and remains close to zero in the non-promoter flanking regions, producing a step-like profile that closely matches the ground-truth binary annotation.

Furthermore, to assess whether this localization ability depends on the fixed 500 bp training window, we extended the evaluation to 1000 bp sequences using a sliding-window scanning strategy (the lower row of Fig 4B). Even in this longer sequence context, the prediction signal remains tightly confined to the annotated promoter region, with minimal false-positive activation in the extended flanking regions. This generalization capacity suggests that the model has learned the latent promoter features rather than memorizing fixed positional patterns tied to the training window size. Although the raw prediction profiles broadly matched the annotated promoter regions, they also showed local discontinuities, with short low-probability gaps embedded within active regions. To address this issue and make the prediction align with biological expectations, a post-processing step was applied to merge nearby predicted segments to obtain coherent promoter annotations. Details of this procedure and additional evaluations on longer DNA sequences are provided in S2 Appendix.

### Motif analysis

To validate the biological plausibility of promoters identified by EvoSNR-Prom, we assessed whether predicted promoter sequences preserve the regulatory motif signatures characteristic of promoters. EvoSNR-Prom was trained on promoter sequences from the circular chromosome of *Agrobacterium tumefaciens* strain C58, whereas all analyses were performed on promoters predicted from the independent linear chromosome.

**Fig 5:**
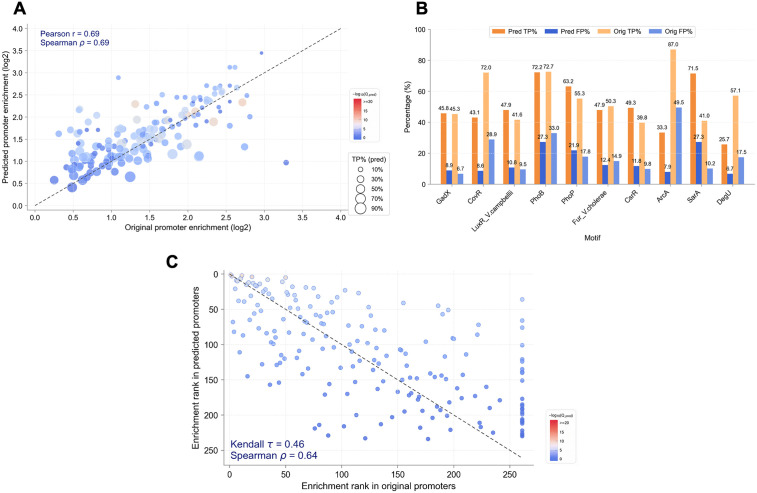
Comparative motif-enrichment analysis. In pannel A and C, each point represents a known transcription factor binding motif. **(A)** Correlation of *log2* enrichment values between predicted and original promoters. The x-axis and y-axis represent the enrichment ratio of the motif, the size of the point represents the proportion of predicted sequences that contain this motif, and the color intensity of the point represents the Q-value. **(B)** Proportions of TP% and FP% for representative motifs. Pred TP% and Orig TP% represent the proportion of the motif in the predicted and original promoter sets, respectively. Pred FP% and Orig FP% denote the proportion of the motif in the predicted and original background sequences, respectively. **(C)** Rank-based correlation of motif relative importance. The x-axis and y-axis indicate the motif enrichment rank in the original and predicted promoter sets, respectively.

First, we compared the regulatory motif landscapes of annotated and predicted promoters using Simple Enrichment Analysis (SEA) [49]. As shown in Fig 5A, motif enrichment ratio exhibited a clear positive correlation (Pearson r = 0.69) between predicted and annotated promoters. Enriched motifs showed highly similar distribution in both annotated and predicted promoters (Fig 5B). Consistently, Fig 5C evaluates rank-level concordance (Spearman ρ = 0.64, Kendall τ = 0.46), confirming that the relative ordering of motif enrichment is preserved. In summary, these results demonstrate that EvoSNR-Prom successfully preserves the global TF binding motif landscape of the original annotated promoter. To further evaluate whether EvoSNR-Prom captures canonical bacterial promoter elements such as -10 bp and -35 bp motifs, we additional analysed σ70-associated −35 and −10 promoter elements and their characteristic spacer lengths. The results are provided in the Supplementary Materials (S3 Appendix), further supporting the biological plausibility of the predicted promoter regions.

Furthermore, to validate the biological relevance of the predicted promoters without relying on predefined motifs, we performed *de novo* motif discovery using MEME tool [37] on the complete set of promoter sequences predicted by EvoSNR-Prom on the test dataset. In parallel, to establish a ground-truth reference, we applied the same discovery process to the annotated promoter regions from the training data. A quantitative comparison of the motifs discovered from the model’s predictions against this reference set was then performed using the TOMTOM algorithm [50]. The discovered motifs showed high similarity to those derived from annotated promoters (S4 Table). In addition, some predicted motifs also matched known regulatory elements in the Combined Prokaryotes Database [51,52] (S4 Table). These findings provide further support that the model can reconstruct core sequence patterns consistent.

## Discussion

In this study, we present EvoSNR-Prom, a novel deep learning framework designed for high-resolution single-nucleotide level prediction of prokaryotic promoters. This framework is built upon the Evo pre-trained model as its backbone and innovatively integrates lexicon enhancement strategy with a label-aware transfer learning module to precisely identify and locate promoter sites within prokaryotic DNA sequences. Experimental results demonstrate that EvoSNR-Prom significantly outperforms current state-of-the-art deep learning models across key performance metrics on multiple datasets. This superior performance not only validates the powerful capability of pre-trained language models in capturing complex DNA sequence patterns but also highlights the effectiveness of our lexicon enhancement and label-aware transfer learning strategies. Furthermore, our analyses of attention weight distributions, transcription factor motif enrichment, and sigma70 factors collectively demonstrate that EvoSNR-Prom’s predictions are not only quantitatively accurate but also biologically interpretable, faithfully recapitulating the regulatory motif landscape of genuine promoters. Consequently, EvoSNR-Prom serves as a reliable computational tool for downstream applications in gene regulatory network analysis and synthetic biology.

Despite these results, certain limitations remain. First, the model’s robustness could benefit from further improvement when processing sequences with complex backgrounds or low signal-to-noise ratios. Performance also tends to decline on longer genomic sequences, where increasing class imbalance and the accumulation of cryptic promoter-like signals in the vast non-promoter background can pose substantial challenges. Second, due to the species-exclusive nature of its training, the model may experience difficulties struggles with direct generalization to novel species outside the training set. To help address these limitations in the future, we aim to construct a large-scale, diverse, pan-prokaryotic promoter dataset to train a universal foundation model with enhanced cross-species generalization capabilities. Additionally, we plan to explore transitioning from a simple classification-based decoder to a more sophisticated, structured prediction mechanism. The current model’s position-wise output layer may not fully capture the inherent dependencies and competitive nature of promoter selection within a local genomic context. We intend to explore advanced decoding architectures, such as Pointer Networks [53] to directly select the single most likely promoter from a set of candidates, or Conditional Random Fields (CRF) [54] to help incorporate grammatical and biological constraints on the entire predicted label sequence. By moving beyond independent, per-nucleotide scoring, this architectural evolution has the potential to empower EvoSNR-Prom to deliver more precise, robust, and biologically plausible predictions, thereby potentially elevating its performance for high-resolution genomic annotation. Finally, expanding the lexicon with species-specific regulatory elements and integrating complementary epigenomic signals [55] could further enhance the model’s discriminative capacity across diverse prokaryotic genomes.

## Supporting information

S1 Table The performance of EvoSNR-Prom in model comparison experiments on *Agrobacterium tumefaciens* strain C58 and *Klebsiella aerogenes* KCTC 2190. (DOCX)

S2 Table Evaluation of the model performance across different source and target data combinations. (DOCX)

S3 Table Examples of attention weight visualization and sequence-level prediction signal (probability) visualization on model input DNA sequences. (DOCX)

S4 Table Comprehensive list of EvoSNR-Prom motif logos comparison. (DOCX)

S1 Appendix Phylogenetic relationships among species and corresponding cross-species transfer performance. (DOCX)

S2 Appendix Post-processing strategy and prediction on longer sequences. (DOCX)

S3 Appendix Evaluation of Canonical σ70 Promoter Features in EvoSNR-Prom Predictions. (DOCX)

S4 Appendix Motif enrichment analysis results across reference motif databases. (DOCX)
