# Supplementary material for: EvoSNR-Prom: Predicting promoters at single-nucleotide resolution with label-aware transfer learning of the pretrained EVO model
Source: PLoS Comput Biol. 2026 Aug 10;22(8):e1014626. doi: 10.1371/journal.pcbi.1014626 (PMC13480636; doi:10.1371/journal.pcbi.1014626)
Supplement: Supplementary file 1 [file pcbi.1014626.s001.docx]

**S1 Table. The Performance of EvoSNR-Prom in model comparison experiments on *Agrobacterium tumefaciens* strain C58 and *Klebsiella aerogenes* KCTC 2190.**

| **DataSet** | **Model** | **Pre**  **（%）** | **F1-score**  **（%）** | **Recall**  **（%）** | **Jaccard**  **（%）** | **MCC**  **（%）** | **AUPRC**  **（%）** |
| --- | --- | --- | --- | --- | --- | --- | --- |
| *Agrobacterium tumefaciens* strain C58 | EvoSNR-Prom | **82.1** | **76.4** | **71.4** | **56.4** | **74.7** | **79.2** |
|  | DeepSNR | 50.7 | 24.8 | 16.4 | 14.1 | 25.6 | 34.2 |
|  | D-AEDNet | 26.0 | 16.5 | 12.1 | 9.8 | 23.4 | 16.8 |
|  | BertSNR | 58.5 | 57.4 | 56.3 | 40.2 | 53.7 | 60.1 |
|  | NTV3(650M post) | 72.8 | 70.7 | 68.7 | 54.6 | 68.2 | 71.6 |
| *Klebsiella aerogenes* KCTC 2190 | EvoSNR-Prom | 90.3 | **84.5** | **79.4** | **69.4** | **82.8** | 83.1 |
|  | DeepSNR | **91.3** | 41.7 | 26.4 | 26.3 | 48.8 | 67.3 |
|  | D-AEDNet | 75.4 | 54.1 | 42.2 | 37.1 | 52.4 | 55.3 |
|  | BertSNR | 73.5 | 71.0 | 68.7 | 55.0 | 67.3 | 80.5 |
|  | NTV3(650M post) | 84.4 | 81.5 | 78.7 | 68.7 | 79.1 | **89.2** |
