# Supplementary material for: EvoSNR-Prom: Predicting promoters at single-nucleotide resolution with label-aware transfer learning of the pretrained EVO model
Source: PLoS Comput Biol. 2026 Aug 10;22(8):e1014626. doi: 10.1371/journal.pcbi.1014626 (PMC13480636; doi:10.1371/journal.pcbi.1014626)
Supplement: Supplementary file 2 [file pcbi.1014626.s002.docx]

**S2 Table. Evaluation of the model performance across different source and target data combinations.**

| **Method** | **Recall** | **F1-score** | **MCC** | **AUPRC** |
| --- | --- | --- | --- | --- |
| Target-only | 70.27% | 68.88% | 66.09% | 71.33% |
| Source-only | 71.33% | 55.48% | 52.20% | 56.11% |
| Source+Target | 72.37% | 72.69% | 69.21% | 72.03% |
