# Supplementary material for: EvoSNR-Prom: Predicting promoters at single-nucleotide resolution with label-aware transfer learning of the pretrained EVO model
Source: PLoS Comput Biol. 2026 Aug 10;22(8):e1014626. doi: 10.1371/journal.pcbi.1014626 (PMC13480636; doi:10.1371/journal.pcbi.1014626)
Supplement: Supplementary file 4 [file pcbi.1014626.s004.docx]

**S4 Table. Comprehensive list of EvoSNR-Prom motif logos comparison.**

| **Motif Logos Comparison of EvoSNR-Prom Predicted Promoters and True Promoters** | | | | | | | | |
| --- | --- | --- | --- | --- | --- | --- | --- | --- |
| **Species: *Agrobacterium tumefaciens* strain C58** | | | | | | | | |
|   P-value:8.57e-05  E-value:6.00e-03 | | | |   P-value: 4.68e-05  E-value: 3.27e-03 | | | |   P-value: 2.81e-05  E-value: 1.97e-03 |
|   P-value: 3.10e-04  E-value: 2.17e-02 | | | |   P-value: 5.63e-04  E-value: 3.94e-02 | | | |   P-value: 3.91e-04  E-value: 2.74e-02 |
| **Species: *Klebsiella aerogenes* KCTC 2190** | | | | | | | | |
|   P-value: 1.50e-04  E-value: 5.99e-03 |   P-value: 6.59e-04  E-value: 2.64e-02 | | | |   P-value: 4.78e-04  E-value: 1.91e-02 | | | |
|   P-value: 1.20e-03  E-value: 4.79e-02 | | |   P-value: 4.00e-04  E-value: 1.60e-02 | | |   P-value: 4.84e-05  E-value: 1.94e-03 | | |
| **Motif logos Comparison of EvoSNR-Prom Predicted Promoters and Combined Prokaryotes Database** | | | | | | | | |
| **Species: *Agrobacterium tumefaciens* strain C58** | | | | | | | | |
| Name: RghR    P-value: 1.86e-04  E-value: 4.99e-02 | | Name: Sigma28    P-value: 1.61e-04  E-value: 4.32e-02 | | | | | Name: DnaA    P-value: 1.15e-05  E-value: 3.09e-03 | |
| **Species: *Klebsiella aerogenes* KCTC 2190** | | | | | | | | |
| Name:PtxR    P-value: 2.11e-04  E-value: 5.69e-02 | | | | Name: MurR    P-value: 1.05e-04  E-value: 2.82e-02 | | | | Name: RhaR    P-value: 1.26e-04  E-value: 3.40e-02 |
