# Supplementary material for: EvoSNR-Prom: Predicting promoters at single-nucleotide resolution with label-aware transfer learning of the pretrained EVO model
Source: PLoS Comput Biol. 2026 Aug 10;22(8):e1014626. doi: 10.1371/journal.pcbi.1014626 (PMC13480636; doi:10.1371/journal.pcbi.1014626)
Supplement: Supplementary file 5 [file pcbi.1014626.s005.docx]

**S1 Appendix. Phylogenetic relationships among species and corresponding cross-species transfer performance.**

**Fig A. Phylogenetic timetree and estimated divergence times of four species generated by TimeTree 5.**

This tree illustrates the divergence times and evolutionary distances among *Agrobacterium tumefaciens*, *Sinorhizobium meliloti* 1021, *Escherichia coli*, and *Klebsiella aerogenes*. Horizontal branch lengths are proportional to absolute evolutionary time, scaled in million years ago (MYA) and mapped against the geologic timescale.

*A. tumefaciens* and *S. meliloti* 1021 split approximately 1200–1400 (MYA). *E. coli* and *K. aerogenes* share a node very close, indicating a highly recent divergence event and minimal evolutionary distance between these sister tax.

**Table A.** **Comparison of model performance on target domain *Agrobacterium tumefaciens* str. C58 trained from diverse source domains.**

| **Source** | **Recall** | **F1-score** | **MCC** | **AUPRC** |
| --- | --- | --- | --- | --- |
| *Escherichia coli str K-12*  substr. MG1655 | 67.50% | 71.61% | 69.43% | 71.68% |
| *Klebsiella aerogenes*  KCTC 2190 | **72.44%** | 69.09% | 66.32% | 71.84% |
| *Sinorhizobium meliloti* 1021 | 71.40% | **76.38%** | **74.66%** | **79.21%** |
