# Supplementary material for: EvoSNR-Prom: Predicting promoters at single-nucleotide resolution with label-aware transfer learning of the pretrained EVO model
Source: PLoS Comput Biol. 2026 Aug 10;22(8):e1014626. doi: 10.1371/journal.pcbi.1014626 (PMC13480636; doi:10.1371/journal.pcbi.1014626)
Supplement: Supplementary file 6 [file pcbi.1014626.s006.docx]

**S2 Appendix. Post-processing strategy and prediction on longer sequences**

1. **Post-processing strategy**

As shown in main manuscript Fig 4B, the raw nucleotide-level prediction profiles sometimes contained fragmented signals, where short low-probability gaps appeared within otherwise continuous promoter-like regions. This is consistent with the biological property of promoter sequences: core promoter elements often provide strong sequence signals, whereas flanking or structurally variable regions may show weaker signals. Therefore, we applied a simple post-processing procedure to convert fluctuating raw predictions into more coherent promoter annotations.

The post-processing procedure consisted of the following steps:

1. Merging adjacent positive segments.

Adjacent promoter-positive segments separated by short background gaps of no more than 10 bp were merged. Specifically, the labels of the intervening gap nucleotides were changed from background to promoter-positive.

2. Filtering short positive segments.

Promoter-positive segments shorter than 30 bp were relabeled as background. This step did not remove any sequence from the input; it only modified the predicted labels to suppress isolated short positive predictions.

**Table A. Performance comparison with and without post-processing on 500 bp and 1000 bp sequences.**

| **Methods** | **500bp** | | **1000bp** | |
| --- | --- | --- | --- | --- |
|  | F1-score | MCC | F1-score | MCC |
| w/o postprocessing | 73.76% | 70.10% | 63.31% | 60.90% |
| postprocessing | 74.68% | 70.98% | 63.64% | 61.34% |

This post-processing strategy was designed to smooth discontinuous promoter-like signals while avoiding over-fragmented annotations. As shown in Table A, post-processing improved performance on both tested sequence lengths. For 500-bp sequences, the F1-score increased from 73.76% to 74.68%, and MCC increased from 70.10% to 70.98%. For 1000-bp sequences, the F1-score increased from 63.31% to 63.64%, and MCC increased from 60.90% to 61.34%.

**2. Prediction on longer sequences**

For sequences longer than the model input length, we used a sliding-window scanning strategy. The model scanned each sequence using 500-bp windows with a 50-bp stride. Prediction probabilities from overlapping windows were aggregated using the mean value to generate a continuous nucleotide-level prediction profile. The same post-processing procedure described above was then applied to the aggregated profile.

The model was evaluated on fixed genomic segments of different lengths, including 500 bp, 1 kb, 10 kb, 20 kb, and 30 kb. As shown in Table B, performance decreased as sequence length increased:

**Table B. Model performance scalability across varying genomic sequence lengths.**

| **Sequence length** | **MCC** | **F1-score** |
| --- | --- | --- |
| 500bp | 70.98% | 74.68% |
| 1kb | 61.34% | 63.64% |
| 10kb | 50.63% | 42.77% |
| 20kb | 38.57% | 29.72% |
| 30kb | 28.88% | 21.28% |

This decline is expected because longer sequences contain substantially larger non-promoter regions, resulting in stronger class imbalance. Even a low nucleotide-level false-positive rate can accumulate over long genomic regions and reduce F1-score and MCC. Nevertheless, the model retained measurable predictive ability on long sequences, suggesting that the sliding-window strategy can extend promoter prediction from fixed-length fragments to broader genomic regions.

In both experiments, EvoSNR-Prom was trained on promoter sequences from the circular chromosome of *Agrobacterium tumefaciens* strain C58, whereas all analyses were performed on promoters predicted from the linear chromosome.
