# Supplementary material for: EvoSNR-Prom: Predicting promoters at single-nucleotide resolution with label-aware transfer learning of the pretrained EVO model
Source: PLoS Comput Biol. 2026 Aug 10;22(8):e1014626. doi: 10.1371/journal.pcbi.1014626 (PMC13480636; doi:10.1371/journal.pcbi.1014626)
Supplement: Supplementary file 7 [file pcbi.1014626.s007.docx]

**S3 Appendix.** **Evaluation of Canonical σ70 Promoter Features in EvoSNR-Prom Predictions**

To further evaluate whether EvoSNR-Prom captures canonical bacterial promoter features, we examined whether the predicted promoters contain the canonical σ70-type core promoter elements, namely the -35 and -10 hexamer boxes, which are major sequence determinants for σ70-dependent transcription initiation in bacteria. This analysis follows the classical bacterial promoter-recognition principle that underlies tools such as BPROM [1]. We scanned each predicted promoter using position-specific scoring matrices PSSMs derived from experimentally verified σ70 promoters in RegulonDB [2]. As shown in Fig A, the sequence logos of the -35 and -10 regions from the RegulonDB reference set (the upper row in Fig A) and those extracted from predicted promoters that passed the PSSM threshold (the lower row in Fig A) exhibit clear conservation patterns (TTGACA-like and TATAAT-like sequences, respectively). In addition, a large proportion of predicted promoters contain both −35 and −10 elements simultaneously above the defined PSSM significance thresholds (Fig B), indicating that many predicted sequences contain a coordinated -35/-10 architecture consistent with canonical σ70-type promoters.

We further analyzed the spacer length distribution between the predicted -35 and -10 boxes (Fig C). Most spacers fell within the optimal 15–19 bp range, with a clear peak at 17 bp, closely matching the canonical spacer distribution reported for experimentally verified σ70 promoters [3]. Together, these results provide strong evidence that EvoSNR-Prom captures key *cis*-regulatory features of prokaryotic promoters.

**Fig A. Comparison of Sequence Logos for RegulonDB and Predicted Sigma70 Promoter -35 and -10 Regions.**

**Fig B. Motif Score Thresholding Analysis Based on -35 and -10 PSSM Scores.** Distribution of PSSM scores for the -35 and -10 boxes across all predicted promoter sequences, and the results of threshold filtering.

**Fig C. Spacer Length Distribution Between -35 and -10 Elements in Predicted Sigma70 Promoters.** Spacer length distribution for the 119 sequences that passed the -35 and -10 box PSSM thresholds.

**References**

1. Solovyev V, Salamov A, Seledtsov I, Vorobyev D, Bachinsky A. Automatic Annotation of Bacterial Community Sequences and Application to Infections Diagnostic. 2011. pp. 346–353.

2. Salgado H, Gama-Castro S, Lara P, Mejia-Almonte C, Alarcón-Carranza G, López-Almazo AG, et al. RegulonDB v12.0: a comprehensive resource of transcriptional regulation in *E. coli* K-12. Nucleic Acids Research. 2024;52: D255–D264. doi:10.1093/nar/gkad1072

3. Cassiano MHA, Silva-Rocha R. Benchmarking Bacterial Promoter Prediction Tools: Potentialities and Limitations.mSystems. 2020 Aug 25; 5(4): e00439-20. doi: 10.1128/mSystems.00439-20.
