# Supplementary material for: EvoSNR-Prom: Predicting promoters at single-nucleotide resolution with label-aware transfer learning of the pretrained EVO model
Source: PLoS Comput Biol. 2026 Aug 10;22(8):e1014626. doi: 10.1371/journal.pcbi.1014626 (PMC13480636; doi:10.1371/journal.pcbi.1014626)
Supplement: Supplementary file 8 [file pcbi.1014626.s008.docx]

**S4 Appendix. Motif enrichment analysis results across reference motif databases.**

The analysis was performed using sequences derived from the linear chromosome of *Agrobacterium tumefaciens* strain C58.The tables are ranked based on statistical significance and summarize motif occurrences across reference databases. Each row represents a candidate motif and includes the following key information: database source (DB), motif identifiers (ID and ALT_ID), consensus sequence (CONSENSUS), enrichment ratio (ENR_RATIO), and statistical significance measured by the false discovery rate-adjusted Q-value (QVALUE). Among these, QVALUE serves as the primary indicator of significance, with lower values indicating higher confidence that the observed enrichment is not due to random chance.

**SEA Annotation Results**

| RANK | DB | ID | ALT_ID | CONSENSUS | ENR_RATIO | QVALUE |
| --- | --- | --- | --- | --- | --- | --- |
| 1 | motif_db/PROKARYOTE/prodoric_2021.9.meme | MX000135 | GadX | WHARRHTWWWWTTWYTWT | 6.56 | 3.13e-20 |
| 2 | motif_db/PROKARYOTE/prodoric_2021.9.meme | MX000038 | CovR | WWWWATTARAR | 2.48 | 1.98e-17 |
| 3 | motif_db/PROKARYOTE/prodoric_2021.9.meme | MX000030 | DegU | AGAAWHHHWHDNHHWTTMDG | 3.24 | 2.02e-16 |
| 4 | motif_db/PROKARYOTE/collectf.meme | EXPREG_00000800 | ArcA_E.coli | WTGTTAAWDAAWTGTTAARWA | 4.17 | 4.99e-16 |
| 5 | motif_db/PROKARYOTE/prodoric_2021.9.meme | MX000091 | ArcA | TGTTAHHWAW | 1.75 | 4.29e-15 |
| 6 | motif_db/PROKARYOTE/prodoric_2021.9.meme | MX000099 | PhoB | YTBTCAKAHAHCTKTVAYHWDH | 2.19 | 4.39e-15 |
| 7 | motif_db/PROKARYOTE/prodoric_2021.9.meme | MX000372 | PhoP | WKWTWWAHDNDTTWWWTA | 3.08 | 4.39e-15 |
| 8 | motif_db/PROKARYOTE/prodoric_2021.9.meme | MX000094 | CysB | TTATTVCHHHWTKNA | 4.42 | 1.6e-14 |
| 9 | motif_db/PROKARYOTE/prodoric_2021.9.meme | MX000335 | H-NS | TATTWWTTT | 2.13 | 1.71e-14 |
| 10 | motif_db/PROKARYOTE/collectf.meme | EXPREG_000008b0 | Fur_V.cholerae | AWTGAKAATNRTTMTCAWTTRNW | 3.33 | 1.71e-14 |
| 11 | motif_db/PROKARYOTE/collectf.meme | EXPREG_00000290 | LuxR_V.campbellii | TTATTGATAWWTTTRTYADTW | 4.28 | 2.84e-14 |
| 12 | motif_db/PROKARYOTE/prodoric_2021.9.meme | MX000236 | SlyA | TTAKVANWTWAA | 2.11 | 4e-14 |
| 13 | motif_db/PROKARYOTE/collectf.meme | EXPREG_00001730 | H-NS_V.cholerae | TCGATAAATT | 1.51 | 5.04e-14 |
| 14 | motif_db/PROKARYOTE/prodoric_2021.9.meme | MX000199 | CovR | TWTTTTAAT | 4.76 | 1.21e-13 |
| 15 | motif_db/PROKARYOTE/prodoric_2021.9.meme | MX000408 | RstA | AWGTTTWMA | 2.61 | 1.26e-13 |
| 16 | motif_db/PROKARYOTE/collectf.meme | EXPREG_00000240 | ToxT_V.cholerae | TRTTTTTTKAWWTY | 4.3 | 1.94e-13 |
| 17 | motif_db/PROKARYOTE/prodoric_2021.9.meme | MX000188 | SarA | ATTWWWADWWAW | 3.96 | 2.78e-13 |
| 18 | motif_db/PROKARYOTE/prodoric_2021.9.meme | MX000366 | CRP | HGHGAYWWWTDTCAMA | 1.74 | 2.78e-13 |
| 19 | motif_db/PROKARYOTE/prodoric_2021.9.meme | MX000096 | Lrp | CAGWAWWHWWTY | 1.62 | 3.05e-13 |
| 20 | motif_db/PROKARYOTE/prodoric_2021.9.meme | MX000041 | CsrR | ATTWKTAMAAAMA | 3.96 | 7.41e-13 |
| 21 | motif_db/PROKARYOTE/prodoric_2021.9.meme | MX000028 | IHF | WAWHHWWTKRWTW | 1.5 | 8.41e-13 |
| 22 | motif_db/PROKARYOTE/collectf.meme | EXPREG_000009a0 | AmpR_P.aeruginosa | HNHATTYKBVAAVCKW | 2.0 | 8.41e-13 |
| 23 | motif_db/PROKARYOTE/prodoric_2021.9.meme | MX000303 | XylR | TTWGTTTAA | 3.79 | 1.23e-12 |
| 24 | motif_db/PROKARYOTE/prodoric_2021.9.meme | MX000128 | Fur | GAWAATNATWATYATTATC | 3.9 | 1.63e-12 |
| 25 | motif_db/PROKARYOTE/prodoric_2021.9.meme | MX000266 | CsgD | AAAAGKGHWAAATW | 1.5 | 1.91e-12 |
| 26 | motif_db/PROKARYOTE/prodoric_2021.9.meme | MX000233 | YrkP | KTYATAWHTT | 1.95 | 1.91e-12 |
| 27 | motif_db/PROKARYOTE/prodoric_2021.9.meme | MX000141 | OmpR | TTKRHAWWYHGATWTAYA | 2.13 | 2.25e-12 |
| 28 | motif_db/PROKARYOTE/prodoric_2021.9.meme | MX000231 | CcpN | TRTKWYATAM | 2.04 | 4.3e-12 |
| 29 | motif_db/PROKARYOTE/prodoric_2021.9.meme | MX000003 | NarL | TACYYMT | 1.49 | 4.3e-12 |
| 30 | motif_db/PROKARYOTE/collectf.meme | EXPREG_00001440 | ExpR_S.meliloti | DMAAADCCCMTVMAWTWWWHKGGGWDN | 2.84 | 4.65e-12 |
| 31 | motif_db/PROKARYOTE/prodoric_2021.9.meme | MX000047 | GerE | AWATAGGYNWWD | 2.13 | 4.65e-12 |
| 32 | motif_db/PROKARYOTE/collectf.meme | EXPREG_00001030 | CRP_V.vulnificus | DTNDYGTGATMAVTNWCANAWWTKN | 2.95 | 5.31e-12 |
| 33 | motif_db/PROKARYOTE/prodoric_2021.9.meme | MX000276 | AmpR | TSTKCTBVAAANTT | 1.43 | 6.96e-12 |
| 34 | motif_db/PROKARYOTE/prodoric_2021.9.meme | MX000206 | AbrB | RNWWAYCCATWWTTTTSAA | 2.52 | 6.96e-12 |
| 35 | motif_db/PROKARYOTE/regtransbase.meme | Crp_Gammaproteobacteria | nan | WWWTGTGAHNYNNMTCACAWWW | 3.48 | 7.96e-12 |
| 36 | motif_db/PROKARYOTE/prodoric_2021.9.meme | MX000346 | MgrA | WYAAHW | 1.79 | 1.88e-11 |
| 37 | motif_db/PROKARYOTE/prodoric_2021.9.meme | MX000118 | CynR | ATAAGKWAA | 3.01 | 4e-11 |
| 38 | motif_db/PROKARYOTE/prodoric_2021.9.meme | MX000174 | SoxS | ATNGCACDNDWKRBYAAHHB | 1.49 | 4.33e-11 |
| 39 | motif_db/PROKARYOTE/collectf.meme | EXPREG_00000c80 | Fur_P.aeruginosa | GHKAATGAWWATCATTWTC | 2.75 | 6.71e-11 |
| 40 | motif_db/PROKARYOTE/prodoric_2021.9.meme | MX000103 | ExsA | TNAAAAAA | 2.75 | 6.71e-11 |
| 41 | motif_db/PROKARYOTE/prodoric_2021.9.meme | MX000029 | Fis | GNHBAWWWWWTVVVC | 1.24 | 1.25e-10 |
| 42 | motif_db/PROKARYOTE/prodoric_2021.9.meme | MX000196 | RghR | RTTGAC | 1.99 | 1.58e-10 |
| 43 | motif_db/PROKARYOTE/prodoric_2021.9.meme | MX000089 | XylR | TTAGTTTGWW | 3.44 | 1.69e-10 |
| 44 | motif_db/PROKARYOTE/prodoric_2021.9.meme | MX000116 | ArgR | TGAATWWWWATKCA | 2.05 | 1.76e-10 |
| 45 | motif_db/PROKARYOTE/prodoric_2021.9.meme | MX000254 | Spo0A | TGTHRAA | 1.4 | 2.14e-10 |
| 46 | motif_db/PROKARYOTE/prodoric_2021.9.meme | MX000054 | MntR | WTTTGCWDDAGRSMAACT | 4.46 | 3.02e-10 |
| 47 | motif_db/PROKARYOTE/collectf.meme | EXPREG_00000ec0 | Fur_N.gonorrhoeae | WWDWHRATAANDATWHTYATTTTW | 5.46 | 3.02e-10 |
| 48 | motif_db/PROKARYOTE/prodoric_2021.9.meme | MX000200 | MelR | HTHHSCDKDWANBSDRAT | 2.67 | 3.02e-10 |
| 49 | motif_db/PROKARYOTE/prodoric_2021.9.meme | MX000169 | NarP | TACGBTH | 1.4 | 3.02e-10 |
| 50 | motif_db/PROKARYOTE/collectf.meme | EXPREG_00000050 | PhoP_Y.pestis | HYGTTKAHDWWYTGTTTAWDW | 5.05 | 3.02e-10 |
| 51 | motif_db/PROKARYOTE/collectf.meme | EXPREG_000008e0 | PhoP_S.enterica | HHHTGTTTANNDWBKGTTTAWNWWHND | 4.88 | 3.02e-10 |
| 52 | motif_db/PROKARYOTE/prodoric_2021.9.meme | MX000045 | CodY | AATTTTSHWAWWWTW | 3.16 | 1.1e-09 |
| 53 | motif_db/PROKARYOTE/collectf.meme | EXPREG_00000d10 | CcpA_C.difficile | DAGAAAACGTTTTCWW | 2.68 | 1.38e-09 |
| 54 | motif_db/PROKARYOTE/prodoric_2021.9.meme | MX000051 | IolR | WAAMCAWNAVT | 2.17 | 1.4e-09 |
| 55 | motif_db/PROKARYOTE/prodoric_2021.9.meme | MX000057 | PhoP | TTMACA | 1.41 | 1.4e-09 |
| 56 | motif_db/PROKARYOTE/prodoric_2021.9.meme | MX000360 | HapR | TGATAANTHWBTMA | 2.78 | 1.5e-09 |
| 57 | motif_db/PROKARYOTE/prodoric_2021.9.meme | MX000016 | Fur | ATTWTHTTWAWWAWAAT | 5.7 | 1.52e-09 |
| 58 | motif_db/PROKARYOTE/prodoric_2021.9.meme | MX000373 | RovA | TTATDHWDDTAV | 5.43 | 1.61e-09 |
| 59 | motif_db/PROKARYOTE/prodoric_2021.9.meme | MX000396 | DcuR | TTTTAWKDATTWWAA | 4.82 | 1.7e-09 |
| 60 | motif_db/PROKARYOTE/prodoric_2021.9.meme | MX000058 | PucR | WTDYTTTGKTT | 1.5 | 1.7e-09 |
| 61 | motif_db/PROKARYOTE/prodoric_2021.9.meme | MX000211 | SdpR | TVHAAAT | 1.59 | 1.76e-09 |
| 62 | motif_db/PROKARYOTE/collectf.meme | EXPREG_00000810 | OmpR_S.enterica | TWTGTAACA | 1.95 | 1.76e-09 |
| 63 | motif_db/PROKARYOTE/prodoric_2021.9.meme | MX000384 | NAC | ATTACHDTNHNHTAT | 1.95 | 1.76e-09 |
| 64 | motif_db/PROKARYOTE/prodoric_2021.9.meme | MX000007 | ArgR | TGTCGCRWHNNYGHAA | 1.28 | 1.89e-09 |
| 65 | motif_db/PROKARYOTE/prodoric_2021.9.meme | MX000264 | LexA | TCGAAMAHDTGTTCGA | 1.65 | 1.92e-09 |
| 66 | motif_db/PROKARYOTE/prodoric_2021.9.meme | MX000149 | Nac | ATMAGAAAATC | 2.18 | 2.98e-09 |
| 67 | motif_db/PROKARYOTE/prodoric_2021.9.meme | MX000363 | AphA | RTGCAASWRRWTKMHW | 2.22 | 3.33e-09 |
| 68 | motif_db/PROKARYOTE/prodoric_2021.9.meme | MX000407 | RirA | WWWWMYMKKRTTWW | 2.88 | 3.73e-09 |
| 69 | motif_db/PROKARYOTE/prodoric_2021.9.meme | MX000401 | VarR | TKNBVAVVRDMNA | 1.55 | 4.5e-09 |
| 70 | motif_db/PROKARYOTE/collectf.meme | EXPREG_00001560 | OxyR_P.aeruginosa | DATHRAYDNWMNCTATBVNNNNNNKNVNNNDNHNNNNDKN | 3.68 | 4.57e-09 |
| 71 | motif_db/PROKARYOTE/prodoric_2021.9.meme | MX000393 | Lrp | MDHDWTTTWY | 1.51 | 5.39e-09 |
| 72 | motif_db/PROKARYOTE/collectf.meme | EXPREG_00000370 | Fur_A.ferrooxidans | YSWHAAWATWNTYATYCKYWN | 2.46 | 5.45e-09 |
| 73 | motif_db/PROKARYOTE/prodoric_2021.9.meme | MX000338 | OmpR | TTTVHVAHWNHA | 1.74 | 5.45e-09 |
| 74 | motif_db/PROKARYOTE/prodoric_2021.9.meme | MX000182 | TyrR | TGTAAWKHWAWVTTTACA | 2.16 | 5.77e-09 |
| 75 | motif_db/PROKARYOTE/prodoric_2021.9.meme | MX000134 | ArgP | WMTDAWDW | 1.47 | 6.53e-09 |
| 76 | motif_db/PROKARYOTE/prodoric_2021.9.meme | MX000024 | CcpC | ATAAGW | 1.77 | 7.59e-09 |
| 77 | motif_db/PROKARYOTE/prodoric_2021.9.meme | MX000151 | NhaR | TCRTWWWWDHYVA | 1.38 | 1.02e-08 |
| 78 | motif_db/PROKARYOTE/prodoric_2021.9.meme | MX000322 | CodY | AWWWMAARARASWTWT | 3.32 | 1.28e-08 |
| 79 | motif_db/PROKARYOTE/collectf.meme | EXPREG_00000360 | Fur_A.salmonicida | RTTDWDAATGATWATTATTMWNAWT | 6.24 | 1.38e-08 |
| 80 | motif_db/PROKARYOTE/collectf.meme | EXPREG_00001110 | Fur_P.syringae | WTGMDAATSATTCBCRW | 1.33 | 1.68e-08 |
| 81 | motif_db/PROKARYOTE/prodoric_2021.9.meme | MX000394 | ArsR | TAAATBVKTBTWH | 3.66 | 2.71e-08 |
| 82 | motif_db/PROKARYOTE/collectf.meme | EXPREG_00001520 | CcpA_L.lactis | TGWAADCGYTTWCA | 1.95 | 2.83e-08 |
| 83 | motif_db/PROKARYOTE/collectf.meme | EXPREG_000010c0 | CodY_B.anthracis | WCWGAAWATTC | 2.14 | 3.49e-08 |
| 84 | motif_db/PROKARYOTE/prodoric_2021.9.meme | MX000023 | ComK | AAAAYNNBDTTTT | 2.76 | 3.98e-08 |
| 85 | motif_db/PROKARYOTE/collectf.meme | EXPREG_00000840 | Lrp_E.coli | YRGHWTTTTHTGCTG | 1.54 | 4.04e-08 |
| 86 | motif_db/PROKARYOTE/collectf.meme | EXPREG_00001280 | ArcA_S.oneidensis | GTTAATWAAATGTTA | 4.23 | 4.14e-08 |
| 87 | motif_db/PROKARYOTE/collectf.meme | EXPREG_00001000 | OmpR_Y.pestis | DWWAMDYTTTRTWACATTTW | 5.1 | 4.23e-08 |
| 88 | motif_db/PROKARYOTE/collectf.meme | EXPREG_00000200 | GlnR_S.erythraea | KBMACRNYBSVGAAAC | 1.4 | 4.23e-08 |
| 89 | motif_db/PROKARYOTE/prodoric_2021.9.meme | MX000224 | NsrR | AARATGYAWTT | 2.66 | 4.34e-08 |
| 90 | motif_db/PROKARYOTE/prodoric_2021.9.meme | MX000334 | Fur | AWTAAH | 2.69 | 6.51e-08 |
| 91 | motif_db/PROKARYOTE/prodoric_2021.9.meme | MX000268 | NikR | TATTATWAWTDWWWWWWWWWTAWTA | 4.74 | 1.02e-07 |
| 92 | motif_db/PROKARYOTE/prodoric_2021.9.meme | MX000165 | MarA | ATRGCACRWWWTGCYAAMHN | 2.46 | 1.05e-07 |
| 93 | motif_db/PROKARYOTE/prodoric_2021.9.meme | MX000209 | NtcA | GTANYWAAHDWTAC | 2.54 | 1.21e-07 |
| 94 | motif_db/PROKARYOTE/prodoric_2021.9.meme | MX000106 | NarL | TKKHBWW | 1.44 | 1.32e-07 |
| 95 | motif_db/PROKARYOTE/prodoric_2021.9.meme | MX000343 | CysB | GSWWWT | 1.31 | 1.89e-07 |
| 96 | motif_db/PROKARYOTE/prodoric_2021.9.meme | MX000315 | HspR | CTTDANWNWWWWWRAYTAAAD | 5.32 | 2.11e-07 |
| 97 | motif_db/PROKARYOTE/prodoric_2021.9.meme | MX000358 | SaeR | GTTAAKAHWTAGTTAA | 7.8 | 2.29e-07 |
| 98 | motif_db/PROKARYOTE/prodoric_2021.9.meme | MX000323 | DegU | GTAAK | 1.2 | 2.94e-07 |
| 99 | motif_db/PROKARYOTE/prodoric_2021.9.meme | MX000044 | CitT | WWCAAA | 2.37 | 3.29e-07 |
| 100 | motif_db/PROKARYOTE/prodoric_2021.9.meme | MX000299 | LexA | CGAACAAAHDHGMD | 3.62 | 3.55e-07 |
| 101 | motif_db/PROKARYOTE/prodoric_2021.9.meme | MX000185 | XylR | GAAATAWMHHAAT | 3.18 | 3.77e-07 |
| 102 | motif_db/PROKARYOTE/prodoric_2021.9.meme | MX000406 | NdhR | TGRTTMRRWWWYA | 1.48 | 4.6e-07 |
| 103 | motif_db/PROKARYOTE/prodoric_2021.9.meme | MX000107 | OxyR | ATDGRNWTWCTYAAT | 2.17 | 4.91e-07 |
| 104 | motif_db/PROKARYOTE/prodoric_2021.9.meme | MX000359 | Fur | GATDHWWWTTWWTNDYAWT | 4.29 | 5.04e-07 |
| 105 | motif_db/PROKARYOTE/prodoric_2021.9.meme | MX000411 | UxuR | WGDGTKNNAHMWBDWM | 2.06 | 5.06e-07 |
| 106 | motif_db/PROKARYOTE/collectf.meme | EXPREG_00001020 | Fur_S.enterica | GVWAATGMTWATMATTWTC | 2.84 | 6.35e-07 |
| 107 | motif_db/PROKARYOTE/prodoric_2021.9.meme | MX000331 | CpxR | GTAAAARTRKGKRAA | 6.69 | 7.08e-07 |
| 108 | motif_db/PROKARYOTE/prodoric_2021.9.meme | MX000277 | AlgZ | GCBABNABCV | 1.18 | 9.12e-07 |
| 109 | motif_db/PROKARYOTE/prodoric_2021.9.meme | MX000212 | Abh | TGDTWSWWWTW | 2.35 | 9.32e-07 |
| 110 | motif_db/PROKARYOTE/prodoric_2021.9.meme | MX000261 | CcpA | RRGAAAASGTTTTBTT | 3.2 | 9.83e-07 |
| 111 | motif_db/PROKARYOTE/prodoric_2021.9.meme | MX000010 | IHF | CAABDBWT | 1.21 | 1.13e-06 |
| 112 | motif_db/PROKARYOTE/collectf.meme | EXPREG_000009b0 | LasR_P.aeruginosa | NANCTRHCWGWWYTGVTAGBDNB | 1.42 | 1.61e-06 |
| 113 | motif_db/PROKARYOTE/prodoric_2021.9.meme | MX000324 | BfmR | WVCWWDVYVDTACA | 1.6 | 1.7e-06 |
| 114 | motif_db/PROKARYOTE/prodoric_2021.9.meme | MX000367 | HlyU | ACTARMKTWST | 2.57 | 2.07e-06 |
| 115 | motif_db/PROKARYOTE/prodoric_2021.9.meme | MX000050 | Hpr | AATAWWATT | 2.58 | 2.47e-06 |
| 116 | motif_db/PROKARYOTE/prodoric_2021.9.meme | MX000399 | CusR | ATBMCAAWHTTKTMWT | 6.83 | 3.49e-06 |
| 117 | motif_db/PROKARYOTE/prodoric_2021.9.meme | MX000389 | CRP | TKTKADHWRDHTHWCA | 2.34 | 3.94e-06 |
| 118 | motif_db/PROKARYOTE/prodoric_2021.9.meme | MX000216 | YodB | TACTWTTTKWWAGTA | 6.13 | 4.19e-06 |
| 119 | motif_db/PROKARYOTE/prodoric_2021.9.meme | MX000330 | ArcA | GTTAAWKTA | 3.58 | 4.28e-06 |
| 120 | motif_db/PROKARYOTE/prodoric_2021.9.meme | MX000339 | PhoP | TTWAHNDWYKGTTTAW | 3.78 | 4.95e-06 |
| 121 | motif_db/PROKARYOTE/collectf.meme | EXPREG_000008c0 | CtrA_C.crescentus | STTAAYGBYYKRTTAACCAHGD | 4.2 | 5.63e-06 |
| 122 | motif_db/PROKARYOTE/prodoric_2021.9.meme | MX000031 | PhoP | TRTTTADGKTHKRTTTA | 3.39 | 8.21e-06 |
| 123 | motif_db/PROKARYOTE/prodoric_2021.9.meme | MX000217 | YycF | TGTWAHATARHTGTAAH | 2.96 | 8.21e-06 |
| 124 | motif_db/PROKARYOTE/prodoric_2021.9.meme | MX000404 | CbbR | ATYYAHVDWMBBVAW | 2.49 | 1.08e-05 |
| 125 | motif_db/PROKARYOTE/prodoric_2021.9.meme | MX000256 | CodY | AATTWTYTGAWAMTT | 3.77 | 1.12e-05 |
| 126 | motif_db/PROKARYOTE/prodoric_2021.9.meme | MX000201 | LeuO | WSKATWAWT | 4.43 | 1.27e-05 |
| 127 | motif_db/PROKARYOTE/prodoric_2021.9.meme | MX000342 | SlyA | TTWGSAWKKTAA | 2.1 | 1.36e-05 |
| 128 | motif_db/PROKARYOTE/collectf.meme | EXPREG_00000b50 | Vfr_P.aeruginosa | NYNNNNNWHHRTGTGAWCYRBHYMNCAWTTHN | 2.19 | 1.36e-05 |
| 129 | motif_db/PROKARYOTE/prodoric_2021.9.meme | MX000018 | CtsR | RGTCAAAVWWRGTCAAH | 7.02 | 1.63e-05 |
| 130 | motif_db/PROKARYOTE/prodoric_2021.9.meme | MX000143 | GcvA | RNWTDAWATWAHCTAAT | 5.12 | 2.58e-05 |
| 131 | motif_db/PROKARYOTE/prodoric_2021.9.meme | MX000160 | RcsA-RcsB | TAVGRAAHATCYTD | 2.78 | 2.73e-05 |
| 132 | motif_db/PROKARYOTE/prodoric_2021.9.meme | MX000304 | PenI | TTACAWWTGTAR | 4.49 | 2.76e-05 |
| 133 | motif_db/PROKARYOTE/prodoric_2021.9.meme | MX000362 | ToxT | NATTTTTTDWWWT | 2.65 | 3.9e-05 |
| 134 | motif_db/PROKARYOTE/prodoric_2021.9.meme | MX000020 | AraR | ATTWGTACGTAYAAWT | 6.63 | 4.03e-05 |
| 135 | motif_db/PROKARYOTE/collectf.meme | EXPREG_00000760 | NtcA_N.sp. | GTAKCWDNNRWTAC | 1.43 | 4.16e-05 |
| 136 | motif_db/PROKARYOTE/prodoric_2021.9.meme | MX000371 | CRP | TGAKNKHDATCWCACW | 1.53 | 4.87e-05 |
| 137 | motif_db/PROKARYOTE/collectf.meme | EXPREG_00000bc0 | PerR_B.subtilis | TTATAATTATTATAA | 5.29 | 5.38e-05 |
| 138 | motif_db/PROKARYOTE/collectf.meme | EXPREG_00000b00 | CsgD_E.coli | WAAWRKKRHWAAHTWVN | 3.75 | 5.49e-05 |
| 139 | motif_db/PROKARYOTE/prodoric_2021.9.meme | MX000157 | PdhR | DAYYGGYHWGACMADYT | 4.88 | 5.68e-05 |
| 140 | motif_db/PROKARYOTE/prodoric_2021.9.meme | MX000391 | UlaR | CAAWYTTKKWKTGRTT | 3.9 | 5.68e-05 |
| 141 | motif_db/PROKARYOTE/prodoric_2021.9.meme | MX000375 | RcsA-RcsB | TARGATWAWTCTTA | 4.08 | 5.83e-05 |
| 142 | motif_db/PROKARYOTE/prodoric_2021.9.meme | MX000121 | CytR | TGCAA | 1.52 | 6.03e-05 |
| 143 | motif_db/PROKARYOTE/prodoric_2021.9.meme | MX000108 | PhhR | TGTHAAGTTTDYTTTACG | 5.53 | 0.000112 |
| 144 | motif_db/PROKARYOTE/collectf.meme | EXPREG_00001190 | PhhR_P.putida | NKTGYGGWVAADNTDKCDTTMCWCGWDCN | 1.89 | 0.000113 |
| 145 | motif_db/PROKARYOTE/prodoric_2021.9.meme | MX000314 | HrcA | YTAGCACTCT | 1.95 | 0.000125 |
| 146 | motif_db/PROKARYOTE/prodoric_2021.9.meme | MX000368 | PhoB | WDKTCAYAWAWSBDWMMB | 4.1 | 0.000128 |
| 147 | motif_db/PROKARYOTE/regtransbase.meme | ArcA_Gammaproteobacteria | nan | TAACADWTARTTAAC | 8.45 | 0.000135 |
| 148 | motif_db/PROKARYOTE/prodoric_2021.9.meme | MX000013 | Fur | GAWAATKAWWWTCATTATC | 3.1 | 0.000159 |
| 149 | motif_db/PROKARYOTE/prodoric_2021.9.meme | MX000019 | Spo0A | TTTGTCGAAWRA | 1.83 | 0.000175 |
| 150 | motif_db/PROKARYOTE/prodoric_2021.9.meme | MX000158 | MetR | TGAAAADHHHTCA | 2.79 | 0.000189 |
| 151 | motif_db/PROKARYOTE/prodoric_2021.9.meme | MX000156 | MngR | TAATACRAATWYAA | 3.34 | 0.000211 |
| 152 | motif_db/PROKARYOTE/collectf.meme | EXPREG_00000950 | PhoP_E.coli | HNTTGTTTAKVDWHTGTTTAWVW | 5.85 | 0.00022 |
| 153 | motif_db/PROKARYOTE/prodoric_2021.9.meme | MX000198 | CymR | AAWHCMBABTWDACTTATMGGAATT | 5.85 | 0.00022 |
| 154 | motif_db/PROKARYOTE/prodoric_2021.9.meme | MX000329 | MvaT | TATTSRRWTTWMAKGAAYA | 5.85 | 0.00022 |
| 155 | motif_db/PROKARYOTE/prodoric_2021.9.meme | MX000009 | ResD | TGADWWTWWTWTGWWHMAWRY | 4.74 | 0.000262 |
| 156 | motif_db/PROKARYOTE/prodoric_2021.9.meme | MX000060 | PrfA | WTAACAWWTGTTAA | 4.74 | 0.000262 |
| 157 | motif_db/PROKARYOTE/prodoric_2021.9.meme | MX000184 | PlaR | AADTGAAACDBYGTTTCAHWW | 4.74 | 0.000262 |
| 158 | motif_db/PROKARYOTE/prodoric_2021.9.meme | MX000271 | MogR | TTTTTWWWAAWAA | 4.39 | 0.000268 |
| 159 | motif_db/PROKARYOTE/prodoric_2021.9.meme | MX000388 | CpxR | GTAAABAAKGGHAWA | 7.8 | 0.000332 |
| 160 | motif_db/PROKARYOTE/collectf.meme | EXPREG_00000f90 | GlxR_C.glutamicum | NNNDNTGTGAHBYRNVHCACANHNNN | 1.51 | 0.000332 |
| 161 | motif_db/PROKARYOTE/prodoric_2021.9.meme | MX000361 | ToxR | TGATTTTTDW | 1.75 | 0.000343 |
| 162 | motif_db/PROKARYOTE/prodoric_2021.9.meme | MX000175 | SdiA | AWAAGNDNDTGANGAAAA | 1.91 | 0.000344 |
| 163 | motif_db/PROKARYOTE/collectf.meme | EXPREG_00000c30 | EspR_M.tuberculosis | BCRGCAAAB | 1.38 | 0.000377 |
| 164 | motif_db/PROKARYOTE/prodoric_2021.9.meme | MX000355 | HspR | CTTDATTBAVWDMBAVTHAAK | 6.34 | 0.000427 |
| 165 | motif_db/PROKARYOTE/prodoric_2021.9.meme | MX000387 | RcsA-RcsB | TRAGRTWATTCYY | 2.77 | 0.000438 |
| 166 | motif_db/PROKARYOTE/prodoric_2021.9.meme | MX000093 | CRP | TGTGAWNHDNWTCACA | 2.28 | 0.000521 |
| 167 | motif_db/PROKARYOTE/prodoric_2021.9.meme | MX000245 | FnrL | TTGAYCBRSRTCAA | 3.9 | 0.000557 |
| 168 | motif_db/PROKARYOTE/prodoric_2021.9.meme | MX000390 | PecS | WAWVTHMHSBYMRVBR | 1.57 | 0.000623 |
| 169 | motif_db/PROKARYOTE/collectf.meme | EXPREG_000006f0 | IHF_P.putida | NNBHYKNHSRRGDDNWWCAAVRNBWTGV | 1.84 | 0.000639 |
| 170 | motif_db/PROKARYOTE/prodoric_2021.9.meme | MX000006 | SpoIIID | WARVACARBC | 1.6 | 0.000657 |
| 171 | motif_db/PROKARYOTE/collectf.meme | EXPREG_000009c0 | CcpA_B.subtilis | HHWWWWWTGWAAGCGYTTTCWWWWWWD | 2.99 | 0.000693 |
| 172 | motif_db/PROKARYOTE/prodoric_2021.9.meme | MX000320 | CzcR | ATTAC | 1.19 | 0.000724 |
| 173 | motif_db/PROKARYOTE/prodoric_2021.9.meme | MX000139 | MalT | GGRGGDKRBD | 1.21 | 0.000784 |
| 174 | motif_db/PROKARYOTE/prodoric_2021.9.meme | MX000014 | HP166 | AAACSMWTMRTT | 2.21 | 0.000885 |
| 175 | motif_db/PROKARYOTE/collectf.meme | EXPREG_000016a0 | Zur_N.meningitidis | TGTTATWMCATAACA | 3.25 | 0.00092 |
| 176 | motif_db/PROKARYOTE/prodoric_2021.9.meme | MX000301 | LexA | CKAMWWMYHYKMCTW | 2.13 | 0.000927 |
| 177 | motif_db/PROKARYOTE/prodoric_2021.9.meme | MX000087 | TreR | SYTGTATATACARG | 5.85 | 0.000985 |
| 178 | motif_db/PROKARYOTE/regtransbase.meme | FadR_Gammaproteobacteria | nan | ADMDVRKHCGRNCMRBY | 1.47 | 0.00114 |
| 179 | motif_db/PROKARYOTE/prodoric_2021.9.meme | MX000260 | CosR | TWTHARNAAAAWTTAWDWTTW | 4.55 | 0.00116 |
| 180 | motif_db/PROKARYOTE/prodoric_2021.9.meme | MX000319 | Cbl | YTYAGMMRWWKWTWYRCWAAR | 4.55 | 0.00116 |
| 181 | motif_db/PROKARYOTE/collectf.meme | EXPREG_000008d0 | MogR_L.monocytogenes | TWTTTTWHAAWAAAW | 2.86 | 0.00128 |
| 182 | motif_db/PROKARYOTE/collectf.meme | EXPREG_00000700 | IHF_P.putida | NDCAWSKYBTTGAW | 1.74 | 0.00149 |
| 183 | motif_db/PROKARYOTE/prodoric_2021.9.meme | MX000012 | LasR | ACCTRSCWRWYYKGBYRGKK | 1.69 | 0.00153 |
| 184 | motif_db/PROKARYOTE/collectf.meme | EXPREG_00001770 | AdpA_S.coelicolor | KGGCSRGAWY | 1.18 | 0.00157 |
| 185 | motif_db/PROKARYOTE/prodoric_2021.9.meme | MX000049 | GltR | ATYYMAAAWTMAGAT | 3.0 | 0.00158 |
| 186 | motif_db/PROKARYOTE/collectf.meme | EXPREG_00001810 | CcpA_S.suis | WWWWGAAAAHKTTTWSAAAAWNW | 2.6 | 0.00161 |
| 187 | motif_db/PROKARYOTE/collectf.meme | EXPREG_00000020 | LexA_C.glutamicum | WTCGAACAWRTKTTCGA | 2.39 | 0.00164 |
| 188 | motif_db/PROKARYOTE/prodoric_2021.9.meme | MX000086 | SinR | TTTGTCGAADRW | 2.01 | 0.00181 |
| 189 | motif_db/PROKARYOTE/prodoric_2021.9.meme | MX000004 | Fnr | TTGATWTWNATCAA | 2.26 | 0.00181 |
| 190 | motif_db/PROKARYOTE/prodoric_2021.9.meme | MX000052 | LevR | TGTWTYAA | 2.26 | 0.00181 |
| 191 | motif_db/PROKARYOTE/regtransbase.meme | SssR_Proteobacteria | nan | TTKACGTTAACGTAAA | 13.7 | 0.00192 |
| 192 | motif_db/PROKARYOTE/prodoric_2021.9.meme | MX000316 | Cap37 | ATAWDWTHATAWAAAT | 3.47 | 0.00219 |
| 193 | motif_db/PROKARYOTE/prodoric_2021.9.meme | MX000043 | AhrC | MWTKMATTTWTATWMAKR | 5.36 | 0.00219 |
| 194 | motif_db/PROKARYOTE/prodoric_2021.9.meme | MX000400 | YedW | MATBMCAAWHTTKTMWTK | 5.36 | 0.00219 |
| 195 | motif_db/PROKARYOTE/prodoric_2021.9.meme | MX000059 | PurR | AAWWMCGAAYAWTW | 4.23 | 0.00242 |
| 196 | motif_db/PROKARYOTE/prodoric_2021.9.meme | MX000205 | CmeR | TGTAATW | 1.65 | 0.0032 |
| 197 | motif_db/PROKARYOTE/prodoric_2021.9.meme | MX000136 | IlvY | AYRTWKCAAWWWYYGCAATRW | 7.8 | 0.00348 |
| 198 | motif_db/PROKARYOTE/prodoric_2021.9.meme | MX000321 | PhoB | CMKKMASWYYHCAKKMMMAVWS | 7.8 | 0.00348 |
| 199 | motif_db/PROKARYOTE/prodoric_2021.9.meme | MX000336 | HilC | STWTTKTTKKADTWWTHWDMHVAHTAWT | 3.01 | 0.00357 |
| 200 | motif_db/PROKARYOTE/prodoric_2021.9.meme | MX000337 | HilD | STWTTKTTKKADTWWTHWDMHVAHTAWT | 3.01 | 0.00357 |
| 201 | motif_db/PROKARYOTE/prodoric_2021.9.meme | MX000008 | GlpR | GYKCGWWATBGMDCATW | 1.58 | 0.00367 |
| 202 | motif_db/PROKARYOTE/prodoric_2021.9.meme | MX000110 | Vfr | TGTGAWCTRBTKMHCM | 1.26 | 0.00419 |
| 203 | motif_db/PROKARYOTE/prodoric_2021.9.meme | MX000123 | DeoR | TGTTAGAATNCTAWCA | 3.25 | 0.00427 |
| 204 | motif_db/PROKARYOTE/prodoric_2021.9.meme | MX000281 | LexA | CTGTATAAATATACAG | 5.85 | 0.00439 |
| 205 | motif_db/PROKARYOTE/prodoric_2021.9.meme | MX000305 | ArgR | TTTATCATAATTATTCATT | 5.85 | 0.00439 |
| 206 | motif_db/PROKARYOTE/prodoric_2021.9.meme | MX000204 | MurR | ATGATTCC | 1.95 | 0.00452 |
| 207 | motif_db/PROKARYOTE/prodoric_2021.9.meme | MX000392 | GadW | WYMKGWTTWYGWTAYTWW | 3.41 | 0.00453 |
| 208 | motif_db/PROKARYOTE/prodoric_2021.9.meme | MX000098 | DnaA | TTATCCACA | 1.66 | 0.00454 |
| 209 | motif_db/PROKARYOTE/prodoric_2021.9.meme | MX000243 | AmtR | ATCTATAGM | 1.66 | 0.00454 |
| 210 | motif_db/PROKARYOTE/prodoric_2021.9.meme | MX000386 | Fur | RMNAAWRAWWATCATTAWY | 2.65 | 0.00455 |
| 211 | motif_db/PROKARYOTE/collectf.meme | EXPREG_00000130 | HapR_V.cholerae | TTATTGAKWATWWTGTBABTWD | 3.62 | 0.00473 |
| 212 | motif_db/PROKARYOTE/regtransbase.meme | DeoR_Enterobacteriales | nan | TGTKAGAATMNTAACA | 4.29 | 0.00496 |
| 213 | motif_db/PROKARYOTE/prodoric_2021.9.meme | MX000140 | LexA | CTGTATAWAHATMCAG | 2.7 | 0.0051 |
| 214 | motif_db/PROKARYOTE/prodoric_2021.9.meme | MX000259 | Fur | TGCAWN | 1.17 | 0.00512 |
| 215 | motif_db/PROKARYOTE/regtransbase.meme | NagQ_a_b_g_Proteobacteria | nan | TGGTATTDTTTTGGTATT | 11.7 | 0.00515 |
| 216 | motif_db/PROKARYOTE/prodoric_2021.9.meme | MX000154 | OxyR | ATWGATTWHAMCTAT | 2.76 | 0.0057 |
| 217 | motif_db/PROKARYOTE/prodoric_2021.9.meme | MX000248 | DnrF | YTGAYSWMKDHMAW | 1.89 | 0.00698 |
| 218 | motif_db/PROKARYOTE/prodoric_2021.9.meme | MX000190 | FnrL | TTGATCCDSRTCAA | 2.13 | 0.00757 |
| 219 | motif_db/PROKARYOTE/prodoric_2021.9.meme | MX000104 | FleQ | GTCRRWAAATTGAC | 1.84 | 0.00802 |
| 220 | motif_db/PROKARYOTE/prodoric_2021.9.meme | MX000257 | Fur | AAWTGAKAAWMWWWWTYAA | 6.83 | 0.00837 |
| 221 | motif_db/PROKARYOTE/collectf.meme | EXPREG_00000120 | LexA_P.difficile | ARGAACTWWTGTTYDR | 3.17 | 0.00862 |
| 222 | motif_db/PROKARYOTE/prodoric_2021.9.meme | MX000090 | Zur | AATCGTAATSATTTCGAT | 3.17 | 0.00862 |
| 223 | motif_db/PROKARYOTE/prodoric_2021.9.meme | MX000244 | CtrA | TTAAYKVRSCVTTAAC | 1.66 | 0.00877 |
| 224 | motif_db/PROKARYOTE/collectf.meme | EXPREG_000016e0 | RpoN_V.cholerae | TGGCAYRNWWWTTGC | 1.4 | 0.00934 |
| 225 | motif_db/PROKARYOTE/regtransbase.meme | NarP_Gammaproteobacteria | nan | TAMCWCTWAAGAGGTA | 5.2 | 0.00975 |
| 226 | motif_db/PROKARYOTE/prodoric_2021.9.meme | MX000187 | LsrR | AAMAMTYRCMTKMAARAYTK | 5.2 | 0.00975 |
| 227 | motif_db/PROKARYOTE/prodoric_2021.9.meme | MX000246 | DnrD | TTRACSHWGGTYAA | 5.2 | 0.00975 |
| 228 | motif_db/PROKARYOTE/prodoric_2021.9.meme | MX000253 | Spo0A | TGTMGAA | 1.19 | 0.0101 |
| 229 | motif_db/PROKARYOTE/prodoric_2021.9.meme | MX000048 | GltC | ATMTMRWTTWKADAW | 2.66 | 0.0115 |
| 230 | motif_db/PROKARYOTE/prodoric_2021.9.meme | MX000131 | EmrR | GWCRGCWAT | 1.38 | 0.0115 |
| 231 | motif_db/PROKARYOTE/prodoric_2021.9.meme | MX000383 | NifA | TGTBRNNWMKRNSACA | 1.74 | 0.0116 |
| 232 | motif_db/PROKARYOTE/prodoric_2021.9.meme | MX000002 | Anr | TTGAYNBGVATCAA | 1.95 | 0.0123 |
| 233 | motif_db/PROKARYOTE/prodoric_2021.9.meme | MX000152 | NtrC | GCMCBATVDTGGY | 1.15 | 0.0123 |
| 234 | motif_db/PROKARYOTE/prodoric_2021.9.meme | MX000370 | YbtA | ACCCGWWWCGGGT | 1.67 | 0.014 |
| 235 | motif_db/PROKARYOTE/prodoric_2021.9.meme | MX000125 | Cra | TGAAWCGWTTMA | 9.75 | 0.014 |
| 236 | motif_db/PROKARYOTE/prodoric_2021.9.meme | MX000137 | LacI | RRYWGTGAGCGSAWMRCAATT | 9.75 | 0.014 |
| 237 | motif_db/PROKARYOTE/prodoric_2021.9.meme | MX000309 | LitR | TGWAYAAWRTYTKTAYAWMKMWTRTAYA | 9.75 | 0.014 |
| 238 | motif_db/PROKARYOTE/regtransbase.meme | RegR_Streptococcus | nan | TKRGTAAACCGGTTTACTTW | 9.75 | 0.014 |
| 239 | motif_db/PROKARYOTE/prodoric_2021.9.meme | MX000210 | NtcA | GTADCARHTRCTAC | 2.93 | 0.0159 |
| 240 | motif_db/PROKARYOTE/prodoric_2021.9.meme | MX000114 | Ada | AATDWDWDMGCAA | 1.95 | 0.0162 |
| 241 | motif_db/PROKARYOTE/prodoric_2021.9.meme | MX000287 | RsaL | TABSMAHYYBDCATV | 1.72 | 0.0177 |
| 242 | motif_db/PROKARYOTE/prodoric_2021.9.meme | MX000302 | OxyR | TCTTCATSDMA | 2.05 | 0.0182 |

**SEA Prediction Results**

| RANK | DB | ID | ALT_ID | CONSENSUS | ENR_RATIO | QVALUE |
| --- | --- | --- | --- | --- | --- | --- |
| 1 | motif_db/PROKARYOTE/prodoric_2021.9.meme | MX000135 | GadX | WHARRHTWWWWTTWYTWT | 5.03 | 3.08e-12 |
| 2 | motif_db/PROKARYOTE/collectf.meme | EXPREG_00000290 | LuxR_V.campbellii | TTATTGATAWWTTTRTYADTW | 4.36 | 1.15e-11 |
| 3 | motif_db/PROKARYOTE/prodoric_2021.9.meme | MX000199 | CovR | TWTTTTAAT | 4.9 | 1.15e-11 |
| 4 | motif_db/PROKARYOTE/prodoric_2021.9.meme | MX000041 | CsrR | ATTWKTAMAAAMA | 4.13 | 1.28e-11 |
| 5 | motif_db/PROKARYOTE/collectf.meme | EXPREG_00000050 | PhoP_Y.pestis | HYGTTKAHDWWYTGTTTAWDW | 3.71 | 1.28e-11 |
| 6 | motif_db/PROKARYOTE/collectf.meme | EXPREG_000008b0 | Fur_V.cholerae | AWTGAKAATNRTTMTCAWTTRNW | 3.81 | 1.58e-10 |
| 7 | motif_db/PROKARYOTE/prodoric_2021.9.meme | MX000038 | CovR | WWWWATTARAR | 3.62 | 1.98e-10 |
| 8 | motif_db/PROKARYOTE/prodoric_2021.9.meme | MX000206 | AbrB | RNWWAYCCATWWTTTTSAA | 3.84 | 2.84e-10 |
| 9 | motif_db/PROKARYOTE/prodoric_2021.9.meme | MX000099 | PhoB | YTBTCAKAHAHCTKTVAYHWDH | 2.63 | 6.02e-10 |
| 10 | motif_db/PROKARYOTE/prodoric_2021.9.meme | MX000372 | PhoP | WKWTWWAHDNDTTWWWTA | 2.86 | 6.02e-10 |
| 11 | motif_db/PROKARYOTE/collectf.meme | EXPREG_00000ec0 | Fur_N.gonorrhoeae | WWDWHRATAANDATWHTYATTTTW | 5.99 | 6.19e-10 |
| 12 | motif_db/PROKARYOTE/prodoric_2021.9.meme | MX000188 | SarA | ATTWWWADWWAW | 2.61 | 8.34e-10 |
| 13 | motif_db/PROKARYOTE/prodoric_2021.9.meme | MX000028 | IHF | WAWHHWWTKRWTW | 4.53 | 1.54e-09 |
| 14 | motif_db/PROKARYOTE/prodoric_2021.9.meme | MX000141 | OmpR | TTKRHAWWYHGATWTAYA | 3.29 | 2.19e-09 |
| 15 | motif_db/PROKARYOTE/prodoric_2021.9.meme | MX000408 | RstA | AWGTTTWMA | 2.79 | 3.17e-09 |
| 16 | motif_db/PROKARYOTE/prodoric_2021.9.meme | MX000303 | XylR | TTWGTTTAA | 4.13 | 5.15e-09 |
| 17 | motif_db/PROKARYOTE/prodoric_2021.9.meme | MX000016 | Fur | ATTWTHTTWAWWAWAAT | 4.45 | 5.73e-09 |
| 18 | motif_db/PROKARYOTE/prodoric_2021.9.meme | MX000360 | HapR | TGATAANTHWBTMA | 2.86 | 1.84e-08 |
| 19 | motif_db/PROKARYOTE/prodoric_2021.9.meme | MX000407 | RirA | WWWWMYMKKRTTWW | 2.96 | 1.84e-08 |
| 20 | motif_db/PROKARYOTE/prodoric_2021.9.meme | MX000134 | ArgP | WMTDAWDW | 3.09 | 1.84e-08 |
| 21 | motif_db/PROKARYOTE/prodoric_2021.9.meme | MX000091 | ArcA | TGTTAHHWAW | 4.11 | 1.89e-08 |
| 22 | motif_db/PROKARYOTE/collectf.meme | EXPREG_00001030 | CRP_V.vulnificus | DTNDYGTGATMAVTNWCANAWWTKN | 3.3 | 1.89e-08 |
| 23 | motif_db/PROKARYOTE/prodoric_2021.9.meme | MX000154 | OxyR | ATWGATTWHAMCTAT | 3.3 | 1.89e-08 |
| 24 | motif_db/PROKARYOTE/prodoric_2021.9.meme | MX000359 | Fur | GATDHWWWTTWWTNDYAWT | 4.27 | 1.89e-08 |
| 25 | motif_db/PROKARYOTE/collectf.meme | EXPREG_00001440 | ExpR_S.meliloti | DMAAADCCCMTVMAWTWWWHKGGGWDN | 3.32 | 1.92e-08 |
| 26 | motif_db/PROKARYOTE/collectf.meme | EXPREG_000008e0 | PhoP_S.enterica | HHHTGTTTANNDWBKGTTTAWNWWHND | 5.13 | 1.92e-08 |
| 27 | motif_db/PROKARYOTE/prodoric_2021.9.meme | MX000050 | Hpr | AATAWWATT | 3.15 | 1.97e-08 |
| 28 | motif_db/PROKARYOTE/regtransbase.meme | Crp_Gammaproteobacteria | nan | WWWTGTGAHNYNNMTCACAWWW | 2.24 | 2.29e-08 |
| 29 | motif_db/PROKARYOTE/prodoric_2021.9.meme | MX000045 | CodY | AATTTTSHWAWWWTW | 3.04 | 2.39e-08 |
| 30 | motif_db/PROKARYOTE/prodoric_2021.9.meme | MX000373 | RovA | TTATDHWDDTAV | 5.87 | 3.13e-08 |
| 31 | motif_db/PROKARYOTE/prodoric_2021.9.meme | MX000096 | Lrp | CAGWAWWHWWTY | 3.7 | 4.3e-08 |
| 32 | motif_db/PROKARYOTE/collectf.meme | EXPREG_00000360 | Fur_A.salmonicida | RTTDWDAATGATWATTATTMWNAWT | 6.34 | 5.55e-08 |
| 33 | motif_db/PROKARYOTE/prodoric_2021.9.meme | MX000315 | HspR | CTTDANWNWWWWWRAYTAAAD | 6.34 | 5.55e-08 |
| 34 | motif_db/PROKARYOTE/prodoric_2021.9.meme | MX000057 | PhoP | TTMACA | 2.22 | 5.7e-08 |
| 35 | motif_db/PROKARYOTE/prodoric_2021.9.meme | MX000174 | SoxS | ATNGCACDNDWKRBYAAHHB | 4.47 | 6.67e-08 |
| 36 | motif_db/PROKARYOTE/prodoric_2021.9.meme | MX000258 | PerR | AATTAATAAATA | 5.23 | 6.67e-08 |
| 37 | motif_db/PROKARYOTE/prodoric_2021.9.meme | MX000396 | DcuR | TTTTAWKDATTWWAA | 5.23 | 6.67e-08 |
| 38 | motif_db/PROKARYOTE/prodoric_2021.9.meme | MX000094 | CysB | TTATTVCHHHWTKNA | 2.16 | 9.9e-08 |
| 39 | motif_db/PROKARYOTE/collectf.meme | EXPREG_00001730 | H-NS_V.cholerae | TCGATAAATT | 2.65 | 1.25e-07 |
| 40 | motif_db/PROKARYOTE/prodoric_2021.9.meme | MX000268 | NikR | TATTATWAWTDWWWWWWWWWTAWTA | 5.29 | 1.37e-07 |
| 41 | motif_db/PROKARYOTE/prodoric_2021.9.meme | MX000009 | ResD | TGADWWTWWTWTGWWHMAWRY | 5.09 | 1.38e-07 |
| 42 | motif_db/PROKARYOTE/prodoric_2021.9.meme | MX000339 | PhoP | TTWAHNDWYKGTTTAW | 3.61 | 1.43e-07 |
| 43 | motif_db/PROKARYOTE/prodoric_2021.9.meme | MX000334 | Fur | AWTAAH | 2.25 | 1.49e-07 |
| 44 | motif_db/PROKARYOTE/prodoric_2021.9.meme | MX000266 | CsgD | AAAAGKGHWAAATW | 2.34 | 1.49e-07 |
| 45 | motif_db/PROKARYOTE/collectf.meme | EXPREG_00001810 | CcpA_S.suis | WWWWGAAAAHKTTTWSAAAAWNW | 2.95 | 1.49e-07 |
| 46 | motif_db/PROKARYOTE/collectf.meme | EXPREG_00000370 | Fur_A.ferrooxidans | YSWHAAWATWNTYATYCKYWN | 2.97 | 1.66e-07 |
| 47 | motif_db/PROKARYOTE/prodoric_2021.9.meme | MX000404 | CbbR | ATYYAHVDWMBBVAW | 2.5 | 1.66e-07 |
| 48 | motif_db/PROKARYOTE/prodoric_2021.9.meme | MX000051 | IolR | WAAMCAWNAVT | 3.37 | 1.66e-07 |
| 49 | motif_db/PROKARYOTE/prodoric_2021.9.meme | MX000211 | SdpR | TVHAAAT | 2.14 | 1.67e-07 |
| 50 | motif_db/PROKARYOTE/prodoric_2021.9.meme | MX000128 | Fur | GAWAATNATWATYATTATC | 3.9 | 1.82e-07 |
| 51 | motif_db/PROKARYOTE/prodoric_2021.9.meme | MX000059 | PurR | AAWWMCGAAYAWTW | 5.36 | 2.39e-07 |
| 52 | motif_db/PROKARYOTE/prodoric_2021.9.meme | MX000231 | CcpN | TRTKWYATAM | 2.12 | 3.41e-07 |
| 53 | motif_db/PROKARYOTE/prodoric_2021.9.meme | MX000256 | CodY | AATTWTYTGAWAMTT | 3.12 | 3.53e-07 |
| 54 | motif_db/PROKARYOTE/prodoric_2021.9.meme | MX000165 | MarA | ATRGCACRWWWTGCYAAMHN | 2.35 | 3.62e-07 |
| 55 | motif_db/PROKARYOTE/collectf.meme | EXPREG_00001000 | OmpR_Y.pestis | DWWAMDYTTTRTWACATTTW | 5.75 | 4.63e-07 |
| 56 | motif_db/PROKARYOTE/collectf.meme | EXPREG_000009a0 | AmpR_P.aeruginosa | HNHATTYKBVAAVCKW | 2.16 | 4.99e-07 |
| 57 | motif_db/PROKARYOTE/prodoric_2021.9.meme | MX000052 | LevR | TGTWTYAA | 2.32 | 5.73e-07 |
| 58 | motif_db/PROKARYOTE/prodoric_2021.9.meme | MX000151 | NhaR | TCRTWWWWDHYVA | 3.27 | 5.89e-07 |
| 59 | motif_db/PROKARYOTE/prodoric_2021.9.meme | MX000201 | LeuO | WSKATWAWT | 2.18 | 6.4e-07 |
| 60 | motif_db/PROKARYOTE/prodoric_2021.9.meme | MX000394 | ArsR | TAAATBVKTBTWH | 2.96 | 8.46e-07 |
| 61 | motif_db/PROKARYOTE/prodoric_2021.9.meme | MX000384 | NAC | ATTACHDTNHNHTAT | 2.07 | 8.46e-07 |
| 62 | motif_db/PROKARYOTE/prodoric_2021.9.meme | MX000031 | PhoP | TRTTTADGKTHKRTTTA | 4.49 | 1.02e-06 |
| 63 | motif_db/PROKARYOTE/prodoric_2021.9.meme | MX000330 | ArcA | GTTAAWKTA | 4.83 | 1.04e-06 |
| 64 | motif_db/PROKARYOTE/prodoric_2021.9.meme | MX000200 | MelR | HTHHSCDKDWANBSDRAT | 2.42 | 1.14e-06 |
| 65 | motif_db/PROKARYOTE/prodoric_2021.9.meme | MX000389 | CRP | TKTKADHWRDHTHWCA | 2.53 | 1.28e-06 |
| 66 | motif_db/PROKARYOTE/prodoric_2021.9.meme | MX000179 | XapR | AAAASTGTATTGG | 7.16 | 1.39e-06 |
| 67 | motif_db/PROKARYOTE/prodoric_2021.9.meme | MX000363 | AphA | RTGCAASWRRWTKMHW | 3.31 | 1.39e-06 |
| 68 | motif_db/PROKARYOTE/prodoric_2021.9.meme | MX000030 | DegU | AGAAWHHHWHDNHHWTTMDG | 3.76 | 1.39e-06 |
| 69 | motif_db/PROKARYOTE/prodoric_2021.9.meme | MX000401 | VarR | TKNBVAVVRDMNA | 2.13 | 1.39e-06 |
| 70 | motif_db/PROKARYOTE/collectf.meme | EXPREG_00001520 | CcpA_L.lactis | TGWAADCGYTTWCA | 2.54 | 1.39e-06 |
| 71 | motif_db/PROKARYOTE/prodoric_2021.9.meme | MX000023 | ComK | AAAAYNNBDTTTT | 2.24 | 1.77e-06 |
| 72 | motif_db/PROKARYOTE/prodoric_2021.9.meme | MX000275 | Zur | TDWWKRAAAWCNTTTTYVRHW | 5.67 | 1.83e-06 |
| 73 | motif_db/PROKARYOTE/collectf.meme | EXPREG_00001020 | Fur_S.enterica | GVWAATGMTWATMATTWTC | 2.82 | 1.83e-06 |
| 74 | motif_db/PROKARYOTE/prodoric_2021.9.meme | MX000216 | YodB | TACTWTTTKWWAGTA | 8.72 | 1.95e-06 |
| 75 | motif_db/PROKARYOTE/prodoric_2021.9.meme | MX000338 | OmpR | TTTVHVAHWNHA | 2.15 | 2.05e-06 |
| 76 | motif_db/PROKARYOTE/prodoric_2021.9.meme | MX000039 | RofA | TTTTSDHRAAMA | 2.99 | 2.15e-06 |
| 77 | motif_db/PROKARYOTE/prodoric_2021.9.meme | MX000233 | YrkP | KTYATAWHTT | 2.75 | 2.73e-06 |
| 78 | motif_db/PROKARYOTE/prodoric_2021.9.meme | MX000393 | Lrp | MDHDWTTTWY | 3.06 | 3.1e-06 |
| 79 | motif_db/PROKARYOTE/prodoric_2021.9.meme | MX000143 | GcvA | RNWTDAWATWAHCTAAT | 6.27 | 3.38e-06 |
| 80 | motif_db/PROKARYOTE/collectf.meme | EXPREG_00000b00 | CsgD_E.coli | WAAWRKKRHWAAHTWVN | 2.52 | 3.85e-06 |
| 81 | motif_db/PROKARYOTE/prodoric_2021.9.meme | MX000103 | ExsA | TNAAAAAA | 4.69 | 4.05e-06 |
| 82 | motif_db/PROKARYOTE/collectf.meme | EXPREG_00000800 | ArcA_E.coli | WTGTTAAWDAAWTGTTAARWA | 4.9 | 4.05e-06 |
| 83 | motif_db/PROKARYOTE/collectf.meme | EXPREG_00000d10 | CcpA_C.difficile | DAGAAAACGTTTTCWW | 3.27 | 6.11e-06 |
| 84 | motif_db/PROKARYOTE/prodoric_2021.9.meme | MX000276 | AmpR | TSTKCTBVAAANTT | 2.04 | 6.13e-06 |
| 85 | motif_db/PROKARYOTE/prodoric_2021.9.meme | MX000149 | Nac | ATMAGAAAATC | 2.15 | 6.29e-06 |
| 86 | motif_db/PROKARYOTE/collectf.meme | EXPREG_00000120 | LexA_P.difficile | ARGAACTWWTGTTYDR | 3.31 | 6.63e-06 |
| 87 | motif_db/PROKARYOTE/prodoric_2021.9.meme | MX000236 | SlyA | TTAKVANWTWAA | 3.05 | 6.83e-06 |
| 88 | motif_db/PROKARYOTE/collectf.meme | EXPREG_00001280 | ArcA_S.oneidensis | GTTAATWAAATGTTA | 4.09 | 7.31e-06 |
| 89 | motif_db/PROKARYOTE/prodoric_2021.9.meme | MX000368 | PhoB | WDKTCAYAWAWSBDWMMB | 5.99 | 7.31e-06 |
| 90 | motif_db/PROKARYOTE/prodoric_2021.9.meme | MX000029 | Fis | GNHBAWWWWWTVVVC | 3.08 | 7.51e-06 |
| 91 | motif_db/PROKARYOTE/prodoric_2021.9.meme | MX000336 | HilC | STWTTKTTKKADTWWTHWDMHVAHTAWT | 4.95 | 8.15e-06 |
| 92 | motif_db/PROKARYOTE/prodoric_2021.9.meme | MX000337 | HilD | STWTTKTTKKADTWWTHWDMHVAHTAWT | 4.95 | 8.15e-06 |
| 93 | motif_db/PROKARYOTE/prodoric_2021.9.meme | MX000182 | TyrR | TGTAAWKHWAWVTTTACA | 2.44 | 9.83e-06 |
| 94 | motif_db/PROKARYOTE/prodoric_2021.9.meme | MX000089 | XylR | TTAGTTTGWW | 3.6 | 1.03e-05 |
| 95 | motif_db/PROKARYOTE/prodoric_2021.9.meme | MX000367 | HlyU | ACTARMKTWST | 3.19 | 1.04e-05 |
| 96 | motif_db/PROKARYOTE/collectf.meme | EXPREG_000016a0 | Zur_N.meningitidis | TGTTATWMCATAACA | 2.68 | 1.09e-05 |
| 97 | motif_db/PROKARYOTE/prodoric_2021.9.meme | MX000118 | CynR | ATAAGKWAA | 3.67 | 1.09e-05 |
| 98 | motif_db/PROKARYOTE/prodoric_2021.9.meme | MX000217 | YycF | TGTWAHATARHTGTAAH | 3.67 | 1.09e-05 |
| 99 | motif_db/PROKARYOTE/collectf.meme | EXPREG_00000c80 | Fur_P.aeruginosa | GHKAATGAWWATCATTWTC | 2.98 | 1.13e-05 |
| 100 | motif_db/PROKARYOTE/prodoric_2021.9.meme | MX000119 | CpxR | GTAAMRNRASGTAA | 2.51 | 1.14e-05 |
| 101 | motif_db/PROKARYOTE/collectf.meme | EXPREG_000000e0 | CcpA_S.pneumoniae | WWWTGAAAACGMTTWCAHWW | 2.7 | 1.21e-05 |
| 102 | motif_db/PROKARYOTE/prodoric_2021.9.meme | MX000093 | CRP | TGTGAWNHDNWTCACA | 2.15 | 1.34e-05 |
| 103 | motif_db/PROKARYOTE/prodoric_2021.9.meme | MX000013 | Fur | GAWAATKAWWWTCATTATC | 4.07 | 1.42e-05 |
| 104 | motif_db/PROKARYOTE/prodoric_2021.9.meme | MX000210 | NtcA | GTADCARHTRCTAC | 2.02 | 1.7e-05 |
| 105 | motif_db/PROKARYOTE/prodoric_2021.9.meme | MX000160 | RcsA-RcsB | TAVGRAAHATCYTD | 2.09 | 1.76e-05 |
| 106 | motif_db/PROKARYOTE/collectf.meme | EXPREG_000010c0 | CodY_B.anthracis | WCWGAAWATTC | 2.15 | 1.76e-05 |
| 107 | motif_db/PROKARYOTE/collectf.meme | EXPREG_00000950 | PhoP_E.coli | HNTTGTTTAKVDWHTGTTTAWVW | 3.49 | 1.83e-05 |
| 108 | motif_db/PROKARYOTE/prodoric_2021.9.meme | MX000198 | CymR | AAWHCMBABTWDACTTATMGGAATT | 8.72 | 1.84e-05 |
| 109 | motif_db/PROKARYOTE/collectf.meme | EXPREG_00000810 | OmpR_S.enterica | TWTGTAACA | 1.94 | 2.29e-05 |
| 110 | motif_db/PROKARYOTE/prodoric_2021.9.meme | MX000212 | Abh | TGDTWSWWWTW | 2.09 | 2.31e-05 |
| 111 | motif_db/PROKARYOTE/prodoric_2021.9.meme | MX000335 | H-NS | TATTWWTTT | 7.41 | 2.34e-05 |
| 112 | motif_db/PROKARYOTE/prodoric_2021.9.meme | MX000010 | IHF | CAABDBWT | 2.56 | 2.54e-05 |
| 113 | motif_db/PROKARYOTE/prodoric_2021.9.meme | MX000184 | PlaR | AADTGAAACDBYGTTTCAHWW | 6.54 | 2.75e-05 |
| 114 | motif_db/PROKARYOTE/collectf.meme | EXPREG_00000130 | HapR_V.cholerae | TTATTGAKWATWWTGTBABTWD | 5.45 | 3.22e-05 |
| 115 | motif_db/PROKARYOTE/prodoric_2021.9.meme | MX000054 | MntR | WTTTGCWDDAGRSMAACT | 5.45 | 3.22e-05 |
| 116 | motif_db/PROKARYOTE/prodoric_2021.9.meme | MX000391 | UlaR | CAAWYTTKKWKTGRTT | 5.45 | 3.22e-05 |
| 117 | motif_db/PROKARYOTE/prodoric_2021.9.meme | MX000316 | Cap37 | ATAWDWTHATAWAAAT | 3.44 | 3.74e-05 |
| 118 | motif_db/PROKARYOTE/prodoric_2021.9.meme | MX000209 | NtcA | GTANYWAAHDWTAC | 2.43 | 4.57e-05 |
| 119 | motif_db/PROKARYOTE/prodoric_2021.9.meme | MX000058 | PucR | WTDYTTTGKTT | 3.17 | 5.09e-05 |
| 120 | motif_db/PROKARYOTE/prodoric_2021.9.meme | MX000169 | NarP | TACGBTH | 2.26 | 5.22e-05 |
| 121 | motif_db/PROKARYOTE/collectf.meme | EXPREG_000001b0 | CodY_L.lactis | AWTTTTCTGAAAATT | 3.89 | 5.82e-05 |
| 122 | motif_db/PROKARYOTE/prodoric_2021.9.meme | MX000018 | CtsR | RGTCAAAVWWRGTCAAH | 4.02 | 6.21e-05 |
| 123 | motif_db/PROKARYOTE/prodoric_2021.9.meme | MX000399 | CusR | ATBMCAAWHTTKTMWT | 5.6 | 6.83e-05 |
| 124 | motif_db/PROKARYOTE/prodoric_2021.9.meme | MX000150 | NagC | BTTAWTTHAYBWTBCRAAAWAWB | 4.84 | 7.15e-05 |
| 125 | motif_db/PROKARYOTE/prodoric_2021.9.meme | MX000322 | CodY | AWWWMAARARASWTWT | 3.39 | 7.77e-05 |
| 126 | motif_db/PROKARYOTE/prodoric_2021.9.meme | MX000254 | Spo0A | TGTHRAA | 1.77 | 7.77e-05 |
| 127 | motif_db/PROKARYOTE/prodoric_2021.9.meme | MX000362 | ToxT | NATTTTTTDWWWT | 3.03 | 8.04e-05 |
| 128 | motif_db/PROKARYOTE/prodoric_2021.9.meme | MX000003 | NarL | TACYYMT | 1.83 | 8.97e-05 |
| 129 | motif_db/PROKARYOTE/prodoric_2021.9.meme | MX000196 | RghR | RTTGAC | 2.18 | 9.5e-05 |
| 130 | motif_db/PROKARYOTE/prodoric_2021.9.meme | MX000271 | MogR | TTTTTWWWAAWAA | 4.16 | 0.000142 |
| 131 | motif_db/PROKARYOTE/prodoric_2021.9.meme | MX000261 | CcpA | RRGAAAASGTTTTBTT | 3.27 | 0.000147 |
| 132 | motif_db/PROKARYOTE/prodoric_2021.9.meme | MX000388 | CpxR | GTAAABAAKGGHAWA | 4.36 | 0.000147 |
| 133 | motif_db/PROKARYOTE/prodoric_2021.9.meme | MX000044 | CitT | WWCAAA | 1.57 | 0.000184 |
| 134 | motif_db/PROKARYOTE/collectf.meme | EXPREG_00001560 | OxyR_P.aeruginosa | DATHRAYDNWMNCTATBVNNNNNNKNVNNNDNHNNNNDKN | 3.58 | 0.000226 |
| 135 | motif_db/PROKARYOTE/prodoric_2021.9.meme | MX000026 | TnrA | TGTTAVAWWWTMTDACA | 3.69 | 0.000248 |
| 136 | motif_db/PROKARYOTE/collectf.meme | EXPREG_00000b50 | Vfr_P.aeruginosa | NYNNNNNWHHRTGTGAWCYRBHYMNCAWTTHN | 1.95 | 0.000281 |
| 137 | motif_db/PROKARYOTE/prodoric_2021.9.meme | MX000331 | CpxR | GTAAAARTRKGKRAA | 6.1 | 0.000304 |
| 138 | motif_db/PROKARYOTE/prodoric_2021.9.meme | MX000369 | Fur | AATRATAATMATTAYCAWT | 6.1 | 0.000304 |
| 139 | motif_db/PROKARYOTE/prodoric_2021.9.meme | MX000222 | LmrA | AWTATATADVHCGVTCTA | 4.14 | 0.000304 |
| 140 | motif_db/PROKARYOTE/prodoric_2021.9.meme | MX000313 | YxaF | AWTATATADVHCGVTCTA | 4.14 | 0.000304 |
| 141 | motif_db/PROKARYOTE/prodoric_2021.9.meme | MX000260 | CosR | TWTHARNAAAAWTTAWDWTTW | 4.63 | 0.00033 |
| 142 | motif_db/PROKARYOTE/prodoric_2021.9.meme | MX000264 | LexA | TCGAAMAHDTGTTCGA | 2.0 | 0.000338 |
| 143 | motif_db/PROKARYOTE/prodoric_2021.9.meme | MX000098 | DnaA | TTATCCACA | 1.97 | 0.00038 |
| 144 | motif_db/PROKARYOTE/prodoric_2021.9.meme | MX000004 | Fnr | TTGATWTWNATCAA | 2.75 | 0.000461 |
| 145 | motif_db/PROKARYOTE/collectf.meme | EXPREG_00000240 | ToxT_V.cholerae | TRTTTTTTKAWWTY | 3.52 | 0.000476 |
| 146 | motif_db/PROKARYOTE/prodoric_2021.9.meme | MX000012 | LasR | ACCTRSCWRWYYKGBYRGKK | 2.18 | 0.000483 |
| 147 | motif_db/PROKARYOTE/prodoric_2021.9.meme | MX000361 | ToxR | TGATTTTTDW | 1.7 | 0.000502 |
| 148 | motif_db/PROKARYOTE/prodoric_2021.9.meme | MX000190 | FnrL | TTGATCCDSRTCAA | 3.92 | 0.000621 |
| 149 | motif_db/PROKARYOTE/prodoric_2021.9.meme | MX000043 | AhrC | MWTKMATTTWTATWMAKR | 6.54 | 0.000628 |
| 150 | motif_db/PROKARYOTE/prodoric_2021.9.meme | MX000329 | MvaT | TATTSRRWTTWMAKGAAYA | 6.54 | 0.000628 |
| 151 | motif_db/PROKARYOTE/prodoric_2021.9.meme | MX000170 | Rob | ATVGCACRTWWBGYYAAHHW | 1.95 | 0.000672 |
| 152 | motif_db/PROKARYOTE/collectf.meme | EXPREG_00000bc0 | PerR_B.subtilis | TTATAATTATTATAA | 4.67 | 0.000711 |
| 153 | motif_db/PROKARYOTE/collectf.meme | EXPREG_000009b0 | LasR_P.aeruginosa | NANCTRHCWGWWYTGVTAGBDNB | 1.6 | 0.00085 |
| 154 | motif_db/PROKARYOTE/prodoric_2021.9.meme | MX000116 | ArgR | TGAATWWWWATKCA | 2.53 | 0.00102 |
| 155 | motif_db/PROKARYOTE/prodoric_2021.9.meme | MX000371 | CRP | TGAKNKHDATCWCACW | 1.6 | 0.00105 |
| 156 | motif_db/PROKARYOTE/prodoric_2021.9.meme | MX000224 | NsrR | AARATGYAWTT | 2.54 | 0.00118 |
| 157 | motif_db/PROKARYOTE/prodoric_2021.9.meme | MX000346 | MgrA | WYAAHW | 2.56 | 0.00137 |
| 158 | motif_db/PROKARYOTE/prodoric_2021.9.meme | MX000312 | PerR | TTAKAATTATTATAA | 4.09 | 0.00146 |
| 159 | motif_db/PROKARYOTE/prodoric_2021.9.meme | MX000355 | HspR | CTTDATTBAVWDMBAVTHAAK | 5.99 | 0.0015 |
| 160 | motif_db/PROKARYOTE/prodoric_2021.9.meme | MX000048 | GltC | ATMTMRWTTWKADAW | 3.11 | 0.00151 |
| 161 | motif_db/PROKARYOTE/prodoric_2021.9.meme | MX000347 | PerR | YKAKAATSATTYTAA | 3.11 | 0.00151 |
| 162 | motif_db/PROKARYOTE/prodoric_2021.9.meme | MX000123 | DeoR | TGTTAGAATNCTAWCA | 4.36 | 0.00151 |
| 163 | motif_db/PROKARYOTE/prodoric_2021.9.meme | MX000304 | PenI | TTACAWWTGTAR | 5.23 | 0.00155 |
| 164 | motif_db/PROKARYOTE/prodoric_2021.9.meme | MX000323 | DegU | GTAAK | 1.57 | 0.00155 |
| 165 | motif_db/PROKARYOTE/prodoric_2021.9.meme | MX000342 | SlyA | TTWGSAWKKTAA | 1.96 | 0.00165 |
| 166 | motif_db/PROKARYOTE/prodoric_2021.9.meme | MX000386 | Fur | RMNAAWRAWWATCATTAWY | 3.19 | 0.00167 |
| 167 | motif_db/PROKARYOTE/prodoric_2021.9.meme | MX000158 | MetR | TGAAAADHHHTCA | 2.18 | 0.00181 |
| 168 | motif_db/PROKARYOTE/prodoric_2021.9.meme | MX000185 | XylR | GAAATAWMHHAAT | 3.27 | 0.00188 |
| 169 | motif_db/PROKARYOTE/prodoric_2021.9.meme | MX000175 | SdiA | AWAAGNDNDTGANGAAAA | 1.99 | 0.00196 |
| 170 | motif_db/PROKARYOTE/prodoric_2021.9.meme | MX000411 | UxuR | WGDGTKNNAHMWBDWM | 1.7 | 0.002 |
| 171 | motif_db/PROKARYOTE/collectf.meme | EXPREG_00000840 | Lrp_E.coli | YRGHWTTTTHTGCTG | 1.49 | 0.00213 |
| 172 | motif_db/PROKARYOTE/collectf.meme | EXPREG_00000f90 | GlxR_C.glutamicum | NNNDNTGTGAHBYRNVHCACANHNNN | 1.86 | 0.00218 |
| 173 | motif_db/PROKARYOTE/prodoric_2021.9.meme | MX000104 | FleQ | GTCRRWAAATTGAC | 2.46 | 0.0022 |
| 174 | motif_db/PROKARYOTE/collectf.meme | EXPREG_00000700 | IHF_P.putida | NDCAWSKYBTTGAW | 1.86 | 0.00252 |
| 175 | motif_db/PROKARYOTE/prodoric_2021.9.meme | MX000106 | NarL | TKKHBWW | 1.44 | 0.00252 |
| 176 | motif_db/PROKARYOTE/prodoric_2021.9.meme | MX000392 | GadW | WYMKGWTTWYGWTAYTWW | 3.81 | 0.00283 |
| 177 | motif_db/PROKARYOTE/prodoric_2021.9.meme | MX000025 | DinR | CGAACDTDTGTTYG | 3.02 | 0.00306 |
| 178 | motif_db/PROKARYOTE/prodoric_2021.9.meme | MX000390 | PecS | WAWVTHMHSBYMRVBR | 2.01 | 0.00312 |
| 179 | motif_db/PROKARYOTE/prodoric_2021.9.meme | MX000287 | RsaL | TABSMAHYYBDCATV | 2.26 | 0.00322 |
| 180 | motif_db/PROKARYOTE/prodoric_2021.9.meme | MX000140 | LexA | CTGTATAWAHATMCAG | 5.45 | 0.00327 |
| 181 | motif_db/PROKARYOTE/regtransbase.meme | FadR_Gammaproteobacteria | nan | ADMDVRKHCGRNCMRBY | 1.47 | 0.00327 |
| 182 | motif_db/PROKARYOTE/prodoric_2021.9.meme | MX000205 | CmeR | TGTAATW | 1.67 | 0.00332 |
| 183 | motif_db/PROKARYOTE/prodoric_2021.9.meme | MX000019 | Spo0A | TTTGTCGAAWRA | 1.5 | 0.00333 |
| 184 | motif_db/PROKARYOTE/prodoric_2021.9.meme | MX000379 | CRP | TGTGAVBWDVDNYBSND | 2.01 | 0.00355 |
| 185 | motif_db/PROKARYOTE/collectf.meme | EXPREG_00000850 | CRP_E.coli | DWNTGTGAYNSAKWTCACRNTW | 2.37 | 0.00356 |
| 186 | motif_db/PROKARYOTE/prodoric_2021.9.meme | MX000343 | CysB | GSWWWT | 1.4 | 0.0037 |
| 187 | motif_db/PROKARYOTE/prodoric_2021.9.meme | MX000350 | ManR | WWATWAGTAARKKTTAYTWATTA | 13.1 | 0.004 |
| 188 | motif_db/PROKARYOTE/prodoric_2021.9.meme | MX000049 | GltR | ATYYMAAAWTMAGAT | 2.8 | 0.00472 |
| 189 | motif_db/PROKARYOTE/collectf.meme | EXPREG_000006f0 | IHF_P.putida | NNBHYKNHSRRGDDNWWCAAVRNBWTGV | 2.39 | 0.00482 |
| 190 | motif_db/PROKARYOTE/prodoric_2021.9.meme | MX000002 | Anr | TTGAYNBGVATCAA | 2.39 | 0.00482 |
| 191 | motif_db/PROKARYOTE/prodoric_2021.9.meme | MX000263 | GlxR | TGTDWSWKRSYYMRMH | 2.18 | 0.00502 |
| 192 | motif_db/PROKARYOTE/prodoric_2021.9.meme | MX000376 | NodD | ATCCAYRNHATGGAT | 2.85 | 0.0054 |
| 193 | motif_db/PROKARYOTE/prodoric_2021.9.meme | MX000086 | SinR | TTTGTCGAADRW | 2.1 | 0.00582 |
| 194 | motif_db/PROKARYOTE/prodoric_2021.9.meme | MX000320 | CzcR | ATTAC | 1.49 | 0.00602 |
| 195 | motif_db/PROKARYOTE/prodoric_2021.9.meme | MX000060 | PrfA | WTAACAWWTGTTAA | 3.74 | 0.00603 |
| 196 | motif_db/PROKARYOTE/prodoric_2021.9.meme | MX000172 | RhaR | TAYCTTRAATTTTCGACG | 7.63 | 0.00613 |
| 197 | motif_db/PROKARYOTE/prodoric_2021.9.meme | MX000245 | FnrL | TTGAYCBRSRTCAA | 1.99 | 0.00635 |
| 198 | motif_db/PROKARYOTE/prodoric_2021.9.meme | MX000299 | LexA | CGAACAAAHDHGMD | 4.0 | 0.0065 |
| 199 | motif_db/PROKARYOTE/prodoric_2021.9.meme | MX000385 | CitB | GTTTTTYWYKWRKYTWAWTK | 4.0 | 0.0065 |
| 200 | motif_db/PROKARYOTE/prodoric_2021.9.meme | MX000324 | BfmR | WVCWWDVYVDTACA | 1.56 | 0.00655 |
| 201 | motif_db/PROKARYOTE/prodoric_2021.9.meme | MX000400 | YedW | MATBMCAAWHTTKTMWTK | 4.36 | 0.00682 |
| 202 | motif_db/PROKARYOTE/regtransbase.meme | PckR_Rhizobiales | nan | HNWTTWAATCGATTKAAAWN | 4.36 | 0.00682 |
| 203 | motif_db/PROKARYOTE/prodoric_2021.9.meme | MX000314 | HrcA | YTAGCACTCT | 1.83 | 0.00682 |
| 204 | motif_db/PROKARYOTE/collectf.meme | EXPREG_000008d0 | MogR_L.monocytogenes | TWTTTTWHAAWAAAW | 2.62 | 0.00682 |
| 205 | motif_db/PROKARYOTE/prodoric_2021.9.meme | MX000257 | Fur | AAWTGAKAAWMWWWWTYAA | 5.81 | 0.00682 |
| 206 | motif_db/PROKARYOTE/prodoric_2021.9.meme | MX000145 | MetJ | DRACRTCT | 2.97 | 0.00682 |
| 207 | motif_db/PROKARYOTE/prodoric_2021.9.meme | MX000305 | ArgR | TTTATCATAATTATTCATT | 4.9 | 0.00685 |
| 208 | motif_db/PROKARYOTE/prodoric_2021.9.meme | MX000318 | SsuR | BTTWYATBCAGBNTGAAAVHW | 4.9 | 0.00685 |
| 209 | motif_db/PROKARYOTE/prodoric_2021.9.meme | MX000377 | Px | CAAYYCAYRSKKKDDAWDV | 4.9 | 0.00685 |
| 210 | motif_db/PROKARYOTE/prodoric_2021.9.meme | MX000380 | RosR | YRSRAWMTWKSDSWRRAYWYCR | 4.9 | 0.00685 |
| 211 | motif_db/PROKARYOTE/prodoric_2021.9.meme | MX000244 | CtrA | TTAAYKVRSCVTTAAC | 1.86 | 0.00702 |
| 212 | motif_db/PROKARYOTE/collectf.meme | EXPREG_00000870 | RutR_E.coli | TTGACCAKHTGGTCVR | 2.42 | 0.00713 |
| 213 | motif_db/PROKARYOTE/collectf.meme | EXPREG_00000760 | NtcA_N.sp. | GTAKCWDNNRWTAC | 1.89 | 0.00738 |
| 214 | motif_db/PROKARYOTE/collectf.meme | EXPREG_00001110 | Fur_P.syringae | WTGMDAATSATTCBCRW | 1.82 | 0.00758 |
| 215 | motif_db/PROKARYOTE/prodoric_2021.9.meme | MX000382 | Fnr | TTGATMDKSATCAA | 3.05 | 0.00758 |
| 216 | motif_db/PROKARYOTE/prodoric_2021.9.meme | MX000406 | NdhR | TGRTTMRRWWWYA | 2.65 | 0.0076 |
| 217 | motif_db/PROKARYOTE/collectf.meme | EXPREG_000016e0 | RpoN_V.cholerae | TGGCAYRNWWWTTGC | 1.73 | 0.0076 |
| 218 | motif_db/PROKARYOTE/prodoric_2021.9.meme | MX000006 | SpoIIID | WARVACARBC | 3.27 | 0.00992 |
| 219 | motif_db/PROKARYOTE/prodoric_2021.9.meme | MX000024 | CcpC | ATAAGW | 3.27 | 0.00992 |
| 220 | motif_db/PROKARYOTE/regtransbase.meme | CtsR_Firmicutes | nan | RGTCAAARAWRGTCAAA | 10.9 | 0.0107 |
| 221 | motif_db/PROKARYOTE/prodoric_2021.9.meme | MX000136 | IlvY | AYRTWKCAAWWWYYGCAATRW | 10.9 | 0.0107 |
| 222 | motif_db/PROKARYOTE/prodoric_2021.9.meme | MX000365 | IgrB | ATAGTTCGGTCCAAATAT | 10.9 | 0.0107 |
| 223 | motif_db/PROKARYOTE/regtransbase.meme | RegR_Streptococcus | nan | TKRGTAAACCGGTTTACTTW | 10.9 | 0.0107 |
| 224 | motif_db/PROKARYOTE/regtransbase.meme | SssR_Proteobacteria | nan | TTKACGTTAACGTAAA | 10.9 | 0.0107 |
| 225 | motif_db/PROKARYOTE/prodoric_2021.9.meme | MX000125 | Cra | TGAAWCGWTTMA | 1.96 | 0.0107 |
| 226 | motif_db/PROKARYOTE/prodoric_2021.9.meme | MX000191 | PpsR | TGTCARNBMAWDTTGACA | 6.54 | 0.0146 |
| 227 | motif_db/PROKARYOTE/prodoric_2021.9.meme | MX000345 | MetR | TGAAVVWBTYTCA | 6.54 | 0.0146 |
| 228 | motif_db/PROKARYOTE/prodoric_2021.9.meme | MX000409 | FapR | TTAGTAYCWGRTACTAA | 6.54 | 0.0146 |
| 229 | motif_db/PROKARYOTE/collectf.meme | EXPREG_00000200 | GlnR_S.erythraea | KBMACRNYBSVGAAAC | 1.33 | 0.0147 |
| 230 | motif_db/PROKARYOTE/collectf.meme | EXPREG_00000330 | CodY_S.pyogenes | AWTTTTCAGAAAATN | 4.36 | 0.0147 |
| 231 | motif_db/PROKARYOTE/prodoric_2021.9.meme | MX000270 | LexA | AAWAMGAACRTATGTTCGDTT | 4.36 | 0.0147 |
| 232 | motif_db/PROKARYOTE/regtransbase.meme | ArcA_Gammaproteobacteria | nan | TAACADWTARTTAAC | 5.09 | 0.0151 |
| 233 | motif_db/PROKARYOTE/collectf.meme | EXPREG_000008c0 | CtrA_C.crescentus | STTAAYGBYYKRTTAACCAHGD | 5.09 | 0.0151 |
| 234 | motif_db/PROKARYOTE/prodoric_2021.9.meme | MX000087 | TreR | SYTGTATATACARG | 5.09 | 0.0151 |
| 235 | motif_db/PROKARYOTE/prodoric_2021.9.meme | MX000301 | LexA | CKAMWWMYHYKMCTW | 1.89 | 0.016 |
| 236 | motif_db/PROKARYOTE/prodoric_2021.9.meme | MX000090 | Zur | AATCGTAATSATTTCGAT | 3.0 | 0.0183 |
